# Functional Expression of Nicotinic Receptors on iPSC-Derived Astrocytes and Signalling Disturbances by a Panel of Neonicotinoid Pesticides and Their Metabolites

**DOI:** 10.3390/ijms27135902

**Published:** 2026-06-30

**Authors:** Eike Cöllen, Chiara Wolfbeisz, Heidrun Leisner, Karin Grillberger, Jasmin Kormann, Yaroslav Tanaskov, Nadine Dreser, Christiaan Karreman, Thomas Hartung, Gerhard Ecker, Udo Kraushaar, Marcel Leist

**Affiliations:** 1 Doerenkamp-Zbinden Chair for In Vitro Toxicology and Biomedicine, University of Konstanz, 78464 Konstanz, Germany; eike.coellen@uni-konstanz.de (E.C.);; 2 Department of Pharmaceutical Sciences, University of Vienna, 1090 Vienna, Austria; 3 NMI Natural and Medical Sciences Institute, University of Tübingen, 72770 Reutlingen, Germany; 4 Center for Alternatives to Animal Testing, Department of Environmental Health and Engineering, Bloomberg School of Public Health, Johns Hopkins University, Baltimore, MD 21205, USA; 5 Center for Alternatives to Animal Testing in Europe (CAAT-Europe), University of Konstanz, 78457 Konstanz, Germany

**Keywords:** cycloxaprid, flupyradifurone, acetylcholine, alpha-7 receptor, neurotoxicity, desnitroimidacloprid

## Abstract

Little is known about how nicotinic signalling in human astrocytes may contribute to the functional neurotoxicity of compounds related to tobacco alkaloids and neonicotinoid pesticides. We generated a single-cell Ca^2+^-imaging assay in induced pluripotent stem cell (iPSC)-derived astrocytes, and profiled functional expressions of some neurotoxicologically relevant receptors. Responses to pharmacological tool compounds indicated the expression of nicotinic, muscarinic, purinergic, glutamatergic receptors and voltage-gated Na^+^/Ca^2+^ channels. Closer investigation of the nicotinic system, e.g., using the alpha7 nicotinic acetylcholine receptor (nAChR)-selective positive allosteric modulator PNU-120596 and alpha7-preferring agonist (AR-R17779) demonstrated that Ca^2+^ signals elicited by nicotine and neonicotinoids are dominated by alpha7 nAChRs and depend on the downstream activation of L-type Ca^2+^ channels and tetrodotoxin-sensitive Na^+^ channels. Crosstalk of nAChR activation/desensitization was not observed for the inflammatory response elicited by TNF or for activation of glutamatergic or purinergic signalling. However, pre-stimulation of nAChR by neonicotinoids significantly blunted the response to the neurotransmitter acetylcholine. Comparative experiments in the human neuronal cultures (LUHMES cells) revealed similar potency ranges and pharmacological fingerprints for several neonicotinoids and their human-relevant metabolites descyanothiacloprid and desnitroimidacloprid. The pesticide metabolites showed a high potency, compared with their respective parent compounds. After this basic system characterization, the hitherto data-poor pesticides cycloxaprid and flupyradifurone were comparatively profiled in astrocytic and neuronal test systems. They showed the typical features of alpha7 nAChR agonists. The disruption of cholinergic signalling in astrocytes suggests that neonicotinoids affect not only neurons in human brains. Therefore, future neurotoxicity screening approaches may need to consider astrocyte toxicity.

## 1. Introduction

Astrocytes are relatively resistant to biochemical stressors and structural toxicants, but their role in functional neurotoxicity has been little explored. More knowledge on this is relevant, as astrocytes represent 20–40% of the total cell number of human brain areas [1], forming the largest subpopulation of glial cells. They play crucial roles in providing structural support, contributing to organ metabolism and maintaining normal brain functions like ion homeostasis [2,3,4,5,6]. Besides these housekeeping roles, they modulate neuronal signalling, synapse formation [7,8,9,10,11,12,13], and higher brain functions, such as cognition [14,15,16].

Human astrocytes exhibit a greater architectural complexity and cellular pleomorphism than their rodent counterpart. Also, calcium signalling shows species differences [17,18]. When murine brain astrocytes were substituted for their human counterparts, the basal level of excitatory synaptic transmission was enhanced, and cognitive capabilities were improved [16]. An increase in cognitive capabilities by astrocytes is likely due to the modulation of synaptic efficiency of neural circuits [14,19,20,21]. From this, it has been concluded that astrocytic evolution has been crucial to match the increased functional demands throughout hominid evolution [17,18,22,23]. This hypothesis is supported by the transcriptomic studies that found the greatest difference in gene expression in brain between rodents and humans are to be in glial genes [24].

Despite species differences, most studies of astrocytes use murine or rat primary glial cultures [25,26,27,28,29,30,31]. Only a few studies have been performed on primary human astrocytes [32,33], as there is only a limited availability of such cells. Astrocytes, generated from induced pluripotent stem cells (iPSCs) present an alternative model system [34,35,36,37,38,39,40,41,42,43,44]. Until now, they have been rarely used for neurotoxicity studies, although many differentiation protocols have been established [41,45,46,47,48,49,50,51,52,53,54].

Astrocytes have a membrane resting potential of approximately −85 mV [55], while the range can span from −22 to −90 mV according to the brain region, developmental stage, culture conditions, and degree of gap-junction coupling [56]. It has been claimed that, for a given astrocyte, the membrane potential is rather static [55]. The expression of many stabilizing ion channels and the presence of gap junctions between the cells have been considered to prevent rapid voltage jumps. The resultant inability of astrocytes to generate neuron-like action potentials has been considered as evidence that astrocytes are not electrically active [55,57,58]. However, there is ample evidence for dynamic changes of the membrane potential, and of the regulation of astrocytes by typical mechanisms known from neuronal signalling [59,60,61]. It has been shown already in the 1990s that astrocytes can sense glutamatergic transmission by exhibiting increases in cytosolic Ca^2+^ in response to glutamate [62]. Such observations have led to the concept of a tripartite synapse, i.e., involving astrocytes in addition to the pre- and postsynaptic neuron [14,20,63].

A growing body of evidence shows that astrocytes express a wide variety of different receptors and voltage-gated ion channels, and may therefore respond to various stimuli by changes in cytosolic Ca^2+^ [64,65,66,67]. Amongst the prominent receptors that are involved in the regulation of astrocytes are purinergic receptors [25,32,35,68,69] and glutamate receptors [62,70,71,72]. Another important group of receptors are the nicotinic acetylcholine receptors (nAChRs). They form ligand-gated sodium channels, and may thus contribute to excitatory signalling [73,74,75,76,77,78,79]. In particular, the α7 isoform of nAChR is known for its multiple and highly diverse functions in the nervous system. These include roles in physiological functions like cognition and memory and in various neuropathologies [79,80,81,82,83,84,85,86,87,88,89,90,91]. While the main effects of activated nAChR have been associated with neuronal function, α7 receptors are also known to be expressed on astrocytes [28,73,74,75,76,92,93] and they have been shown to be upregulated, e.g., in Alzheimer’s disease (AD) [74,76,94].

Voltage-gated sodium channels (Na_v_) are an important feature of excitable cells, and their subtypes can be distinguished by their sensitivity to the toxicant tetrodotoxin (TTX) [27,66,95,96,97,98]. Several TTX-sensitive (TTX-S) subtypes (Nav1.1, Nav1.2, Nav1.3, Nav1.4, Nav1.5, and Nav1.7) have been found on astrocytes. Some examples of TTX-insensitive Na_v_ (Nav1.5, Nav1.8, and Nav1.9) may also be found [99,100,101,102]. The expression of Na_v_ on astrocytes is affected by culture conditions and the extracellular milieu [27,103,104].

Na^+^ influx into astrocytes (via Na_v_ or nAChR) may be coupled to the increase in intracellular [Ca^2+^] via the activation of voltage-dependent Ca^2+^ channels (VDCCs). These are particularly important for the amplification of the Ca^2+^ influx and modulation of downstream responses in astrocytes [105,106,107,108]. VDCCs are classified as Ca_v_ 1.1–1.4 (L-type Ca^2+^ channels), Ca_v_ 2.1–2.3 (P/Q/N/R-type Ca^2+^ channels), and Ca_v_ 3.1–3.3 (T-type Ca^2+^ channels) [109,110]. Various types of VDCC may be found in cortical astrocyte cultures derived from neonatal rats, but some studies indicate that Ca_v_ 1.2/1.3 dominate the response [106,111,112,113].

Amongst the many neurotransmitter receptors potentially expressed on human astrocytes, the nAChR may play a particularly important toxicological role, as they are the major target of nicotine [31,114,115,116]. Besides their role in the adult brain, nAChR are important for brain development. They are involved in the fine-tuning of the excitation–inhibition equilibrium [117,118,119]. Disturbances of this balance have been associated with attention deficit hyperactivity disorder (ADHD) [120,121,122], autism [123,124,125], and schizophrenia [126,127,128,129,130,131]. In line with this, the nAChR agonist nicotine is well established as a trigger of developmental neurotoxicity (DNT) [31,132,133,134,135,136,137,138].

Besides nicotine, some members of the group of neonicotinoid insecticides (NeoNics) may also trigger nAChR. Such agents have been developed as pesticides, destined to overstimulate the nervous system of insects. Several NeoNics act as agonists also on human nAChR [115,116,139]. In this function, they may have a liability to promote DNT. There are still knowledge gaps on the potency of some members of the neonicotinoid pesticide family, and of some of their major metabolites, for human nAChRs. Moreover, there is little knowledge of the major target cells in the developing nervous system. The main focus has been on neurons, as they develop early during ontogeny and have been considered to be the main players of cognitive processes. With increasing knowledge of the continuing neurodevelopment post birth [140,141,142,143,144] and of the role of astrocytes beyond basic brain homeostasis, it appears important to consider glial cells as targets of nicotinic toxicants. In this study, we aimed to (i) provide a model system for such studies, (ii) to showcase its usefulness as a novel assay platform to study the potency and efficacy of toxicants, and (iii) to provide a comparative case study on NeoNics affecting both astrocytes and neurons.

## 2. Results

### 2.1. Use of iPSC-Derived Astrocytes for the Functional Characterization of ‘Nicotinic Chemicals’

**Figure 1:**
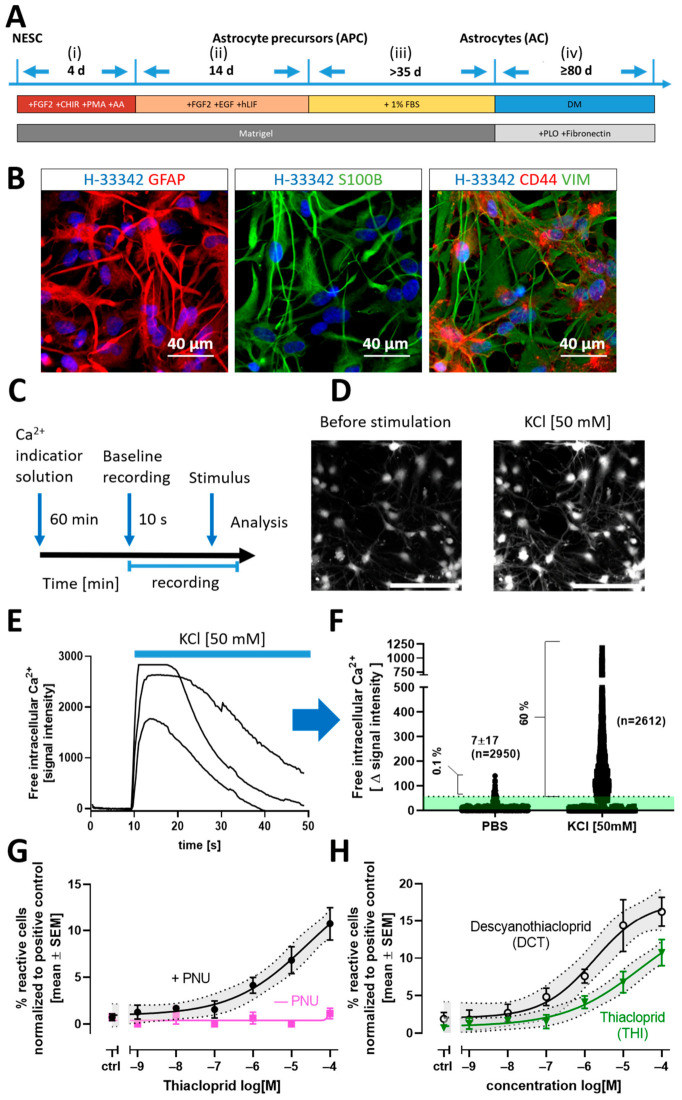
Use of iPSC-derived astrocytes for the functional characterization of nicotinic chemicals. (**A**) Differentiation scheme for iPSC-derived astrocytes: cells were differentiated for 53 days (phases i–iii) and then used for experiments for up to 80 days (phase iv). To allow for comparability with LUHMES neurons, astrocytes were used for experiments in LUHMES differentiation medium (DM) (with cAMP, tetracycline, and glial cell-line-derived neurotrophic factor (GDNF)). (**B**) Immunocytochemistry of astrocyte-specific markers: astrocytes were differentiated for 53 days then plated on Matrigel-coated cover slips at a density of 50,000 cells/cm^2^ in phase iii. The fluorescent signal coupled to the second antibody of glial fibrillary acidic protein (GFAP), S100ß, CD44, and vimentin (VIM) are indicated in the corresponding colour above the pictures. Nuclei were counterstained with H-33342. (**C**) Experimental set-up for the calcium imaging experiments. Astrocytes (iPSC-derived) were plated in 96-well plates and cultured (phase iv) for two days. At 1 h before the experiment, cells were loaded with a Ca^2+^ indicator dye. Test compounds were added by an automated pipettor. Single-cell fluorescence information was recorded for the whole field of view. (**D**) Exemplification of calcium fluorescence images of stage iv astrocytes before and after stimulation with KCl [50 mM], scalebars: 150 µm. (**E**) Individual cell somata were automatically identified by the CaFFEE software and single-cell calcium traces were obtained after stimulation with KCl [50 mM]. (**F**) Threshold determination for the cell population to define a ‘reactive cell’: the average fluorescence (means ± SD) was determined for about 3000 cells. The threshold of ‘reactivity’ was set at ‘means + 3x the standard deviation’ of negative controls. Only 0.1% of solvent-treated cells exceeded that value. Typical data for about 2600 positive controls are shown. (**G**) Concentration response curve of thiacloprid with and without PNU-120596 (PNU), an allosteric modulator of the α7 nicotinic acetylcholine receptor (nAChR). (**H**) Concentration-dependent Ca^2+^ response of thiacloprid (THI) and its metabolite descyanothiacloprid (DCT). Data is presented as % reactive cells, normalized to the number of positive control cells as mean ± SEM of biological triplicates; each biological replicate included three technical replicates. The 90% confidence intervals are indicated by dotted lines and grey background shading. Differences were tested for significance by two-way ANOVA, followed by the Sidak post hoc test for the comparison between treatment ± PNU-120596 and between TIA and DCT. The Gaussian distribution of the residuals was tested with four tests (Anderson–Darling, D’Agostino–Pearson omnibus, Shapiro–Wilk, and Kolmogorvo–Smirnov). The results can the found in Supplementary Materials in the repository https://zenodo.org/records/20840279 (accessed on 25 June 2026).

Astrocytes were generated from iPSC as detailed earlier [54,145]. After about 50 days of differentiation (DoD), cells were mature and could be used for experiments for a period of 2–3 months with essentially similar results (Figure 1A). The majority of cells (>95%) expressed typical astrocytic markers, in particular an elaborate intracellular network of GFAP microfilaments (Figure 1B). For signalling experiments that used the intracellular cytosolic free-Ca^2+^ concentrations ([Ca^2+^]_i_) as endpoint (here abbreviated to ‘Ca^2+^ signalling’ studies), astrocytes were replated, and the medium was exchanged for LUHMES neuron differentiation medium (DM), to allow a direct comparability of astrocytic and neuronal responses (Figure 1A). All experiments involved a loading of cells with the Ca^2+^ indicator dye Cal-520 and a recording of the fluorescence baseline, followed by a quantification of the response to stimulation (Figure 1C,D).

Pilot experiments showed that astrocytes responded to a cell membrane depolarization, triggered by an increase in K^+^ ions in the medium, with a sharp increase in [Ca^2+^]_i_ and a slow return towards the baseline within 20–40 s (Figure 1D,E). The software CaFFEE [146] was used to automatically identify individual cells (based on nuclear staining) and to record Ca^2+^ traces from all identified cells (typically about 20–50 in the imaging area).

To classify cells as responders or non-responders, we used a statistical procedure based on thresholding of the baseline noise [147,148]: astrocytes exposed to solvent (PBS) displayed a fluorescence shift of 7 ± 17 arbitrary units. Using three times the standard deviation of the baseline signal as the threshold, cells with a fluorescence shift of >58 (7 + 3 × 17) were counted as responders. Only 0.1% of astrocytes exposed to solvent alone showed a ‘positive response’, according to this classification scheme.

Cells exposed to KCl [50 mM] showed an average fluorescent shift of 130 ± 166 arbitrary units, and 60% of the astrocyte population were classified as responders (Figure 1F). We considered this assay window, of 60% response between negative and positive controls, sufficiently large and robust for further use in a test method. We also assumed that only cells that responded to KCl were able to respond to other electrophysiological signals linked to Ca^2+^ signalling. Based on this, we normalized all responses to the percentage of KCl responders in the same experiment.

The experimental procedure and the data processing pipeline developed for Ca^2+^ signalling in iPSC-derived astrocyte cultures was used to assess the functional effects of neonicotinoid pesticides. This allowed comparisons with a rich set of reference data available on these compounds for neuronal Ca^2+^ signalling [115]. Studies on neurons had shown that some neonicotinoids (NeoNics) (such as thiacloprid or imidacloprid) triggered Ca^2+^ responses, while others, like thiamethoxam, had no effect on neurons. Such data were essentially confirmed in astrocytes, as imidacloprid (Figure S1A) and thiacloprid showed a clear Ca^2+^ response and thiamethoxam had no effect (Figure S1B).

The neuronal responses to NeoNics are mediated by nicotinic acetylcholine receptors (nAChR) [149]. Amongst the different receptor subtypes, the α7 receptors take a dominant role in human neurons, as Ca^2+^ signalling was only observed in the presence of PNU120596, a selective positive allosteric modulator of α7 nAChRs [115]. Therefore, the standard medium for signalling experiments always contained PNU (unless otherwise indicated). To assess the role of α7 nAChR in astrocytes, we tested the effect of PNU omission from the medium. The NeoNic thiacloprid (THI) showed a concentration-dependent Ca^2+^ signalling response in the presence of PNU with a BMC_10_ of 60.3 µM (C.I. 36.3–97.7 µM), but no response in its absence, with an extrapolated BMC_10_ of 173.8 µM (Figure 1G). Control experiments showed that PNU alone had no effect on [Ca^2+^]_i_. This suggests strongly that α7 nAChRs play a dominant role in astrocytic responses to thiacloprid in the presence of PNU.

In another set of scoping experiments, we explored whether astrocytes would be able to detect and quantify responses to important NeoNic metabolites. Neuronal signalling studies showed the importance of such information, as some metabolites are more potent than the parent compounds and may thus contribute to NeoNic toxicity [116,139]. One such parent–metabolite pair is THI with its human-relevant metabolite descyanothiacloprid (DCT) [139]. In good agreement with neuronal studies, we found that DCT also acts on astrocytes, and triggers a more pronounced response (BMC_10_ of 1.8 µM (C.I. 0.05–67.6 µM)), in a direct comparison with its parent (BMC_10_ of 60.3 µM (C.I. 36.3–97.9 µM) (Figure 1H). Another well-known parent–metabolite pair is imidacloprid (IMI) with its metabolite desnitro-imidacloprid (DN-IMI). Signalling studies in astrocytes confirmed that the metabolite DN-IMI triggers a more pronounced response (BMC_10_ 0.23 µM (C.I. 0.02–3.02 µM)) than its parent compound (BMC_10_ 2.57 µM (C.I. 0.03–251.2 µM)) (Figure S1A).

As the neuronal DM medium used for experiments is serum-free, while astrocytes are maintained in serum-containing medium before the experiments, the responsiveness of the astrocytes in DM with and without 1% serum was tested for the stimulation with THI. No difference in the responsiveness between the two conditions was observed (Figure S1C). Based on this, serum was omitted for all following experiments. Data obtained for THI were confirmed by a small panel of other compounds comparatively tested in astrocytic medium or DM (Figure S1D). Further, we investigated whether the normalization influences the determination of the BMC_10_. The BMCs between the normalized data 0.15 (C.I 0.065–0.35) did not vary from those of the non-normalized data 0.35 (C.I. 0.078–1.51) (Figure S1E). From these scoping experiments, we conclude that (i) testing of functional astrocytic responses, based on changes in [Ca^2+^]_i_ as endpoint, is a robust test method, which (ii) may be applied to environmental agents triggering nAChR, and (iii) that the method can be performed in the same medium as that used for neurons, to allow direct quantitative comparisons of responses.

### 2.2. Comparison of Nicotinic Responses to Other Ca^2+^ Signals That May Be Triggered in Astrocytes

**Figure 2:**
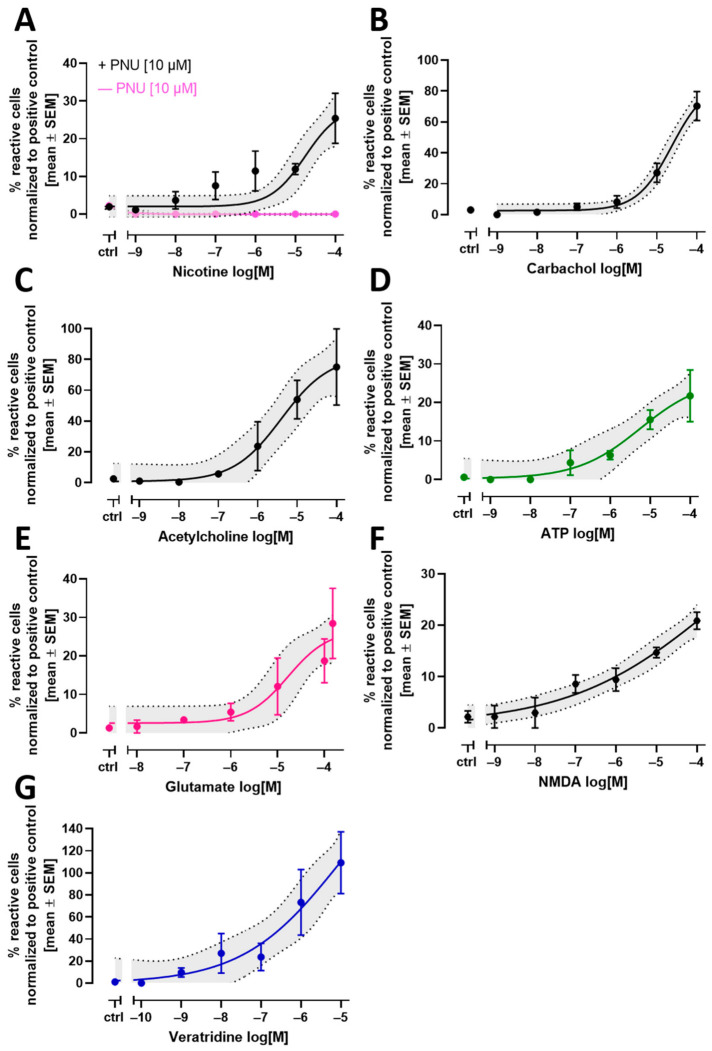
Brief overview of astrocytic Ca^2+^ signalling responses. Astrocytes were used as in Figure 1G. Concentration–response curves were obtained for tool compounds known to specifically trigger various physiological responses in neurons. (**A**) Concentration–response curves for nicotine with and without the allosteric modulator PNU. (**B**) Concentration-dependent reaction to Carbachol, an agonist for muscarinic acetylcholine receptors (mAChRs). (**C**) Concentration–response for acetylcholine (ACh), the physiological ligand of nAChR and mAChR. (**D**) Concentration–response for ATP, an agonist for purinergic receptors. (**E**) Concentration–response for glutamate, an agonist for several classes of glutamate receptors. (**F**) Concentration–response for NMDA, a specific agonist for ionotropic NMDA-class glutamate receptors. (**G**) Concentration-dependent reaction to the Na_v_ agonist veratridine (VTD). Data is presented as % reactive cells normalized to the positive control (KCl). All data are mean ± SEM of biological triplicates each biological replicate included three technical replicates. The 90% confidence intervals are indicated by dotted lines and grey background shading. Differences were tested for significance by two-way ANOVA, followed by Dunnett’s multiple comparison post hoc test for comparison between treatments and control. The Gaussian distribution of the residuals was tested with four tests (Anderson–Darling, D’Agostino–Pearson omnibus, Shapiro–Wilk, and Kolmogorvo–Smirnov). The results can be found in Supplementary Materials in the repository https://zenodo.org/records/20840279 (accessed on 25 June 2026).

After assessment of the response to the NeoNics THI, DCT, IMI, and DN-IMI, we were interested in obtaining information on the archetypical nAChR agonist nicotine (NIC). Its response patterns were assessed with and without the addition of the allosteric modulator PNU. NIC plus PNU triggered a concentration-dependent increase in [Ca^2+^]_i_ in a subpopulation of astrocytes (up to 25% of all responsive cells) (BMC_10_ 1.15 µM (C.I. 0.28–4.68 µM)), while NIC without the addition of PNU did not evoke any response (Figure 2A). This is in good concordance with the data obtained from LUHMES neurons in previous studies [115].

Next, we explored whether responses of muscarinic acetylcholine receptors (mAChR) may also be assessed by our test method. The specific agonist carbachol was used to activate these metabotropic receptors. It evoked a response at concentrations ≥ 10 µM and reached a maximum (70% of responsive cells) at about 100 µM (BMC_10_ 1.78 µM (C.I. 0.87–3.63 µM) (Figure 2B). As a next step, we tested acetylcholine (ACh), the physiological ligand for both types of AChRs (metabotropic mAChR and ionotropic nAChR). Astrocytes were treated with increasing concentrations of ACh, which started to evoke a response in astrocytes at ≥1 µM (BMC_10_ 0.15 µM (C.I. 0.06–0.35 µM) (Figure 2C). At the highest tested concentration (HTC), ACh triggered [Ca^2+^]_i_ changes in 75% of the stimulable astrocytes. In neuronal LUHMES cultures, only about 45% of the cells reacted to ACh [115].

To broaden the background information on the astrocyte test system, some other candidate receptors were considered. First, we tested for purinergic receptors, well known for their astrocytic roles [35,68,69]. We used the physiological ligand adenosinetriphosphate (ATP). It evoked a response at ≥0.1 µM, and, at the HTC (100 µM), altogether 22% of astrocytes responded (BMC_10_ 3.39 µM (C.I. NaN) (Figure 2D). This situation was different from LUHMES neurons, where ATP triggered Ca^2+^ signals in 100% of cells at 100 µM [150].

Secondly, we considered the glutamate receptor family [151,152]. Glutamate evoked a concentration-dependent response at ≥10 µM, and it reached a maximum of 28% responders at the HTC of 150 µM (BMC_10_ 6.03 µM (C.I. 1.17–31.62 µM) (Figure 2E). N-metyhl-D-aspartate (NMDA) triggered a response at ≥0.1 µM, with a number of 21% responders at the HTC of 100 µM (BMC_10_ 1.17 µM (C.I. 0.47–3.02 µM) (Figure 2F). These data suggest that a subpopulation of our human astrocytes is highly sensitive to NMDA. The complexity of all different GluR signals is beyond the scope of this work, but may be worth exploring in the future. This may also be relevant for comparisons with LUHMES neuronal cultures, where no information on glutamate receptor signalling is available yet, although a variety of different subunits of the glutamate receptor family (both metabotropic and ionotropic receptor subtypes) appears to be expressed [150].

As the final part of our general characterization study, voltage-gated sodium channels (VGSCs) were explored [153]. The universal VGSCs activator veratridine (VTD) was used [154]. It evoked an increase in [Ca^2+^]_i_ in astrocytes at ≥0.01 µM with 100% responders at the HTC of 10 µM (BMC_10_ 0.01 µM (C.I. 0.00006–2.09 µM) (Figure 2G). These findings were comparable to those obtained in LUHMES neurons [150], and they showed that all excitable astrocytes expressed a sizable number of VGSCs.

In summary, these results indicate that the test system of iPSC-derived astrocytes can be used to study a wide variety of different receptors for toxicological approaches. When focussing on a particular receptor type or toxicant class, control experiments with selective inhibitors and modulators are advisable, to ensure response specificity.

### 2.3. Pharmacological Profiling of Functional nAChR Receptor Expression in hiPSC-Derived Astrocytes

**Figure 3:**
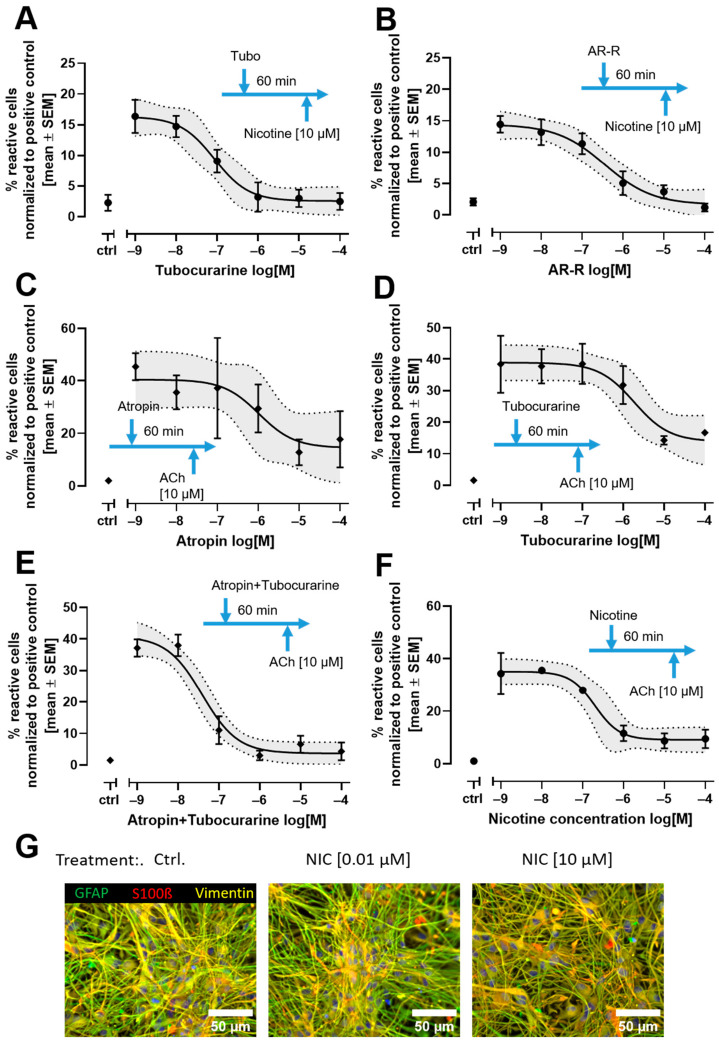
Functional nAChR expression in hiPSC-derived astrocytes. Functional nAChR expression in hiPSC-derived astrocytes was investigated by modulation of the response of the nAChR with known tool compounds in calcium imaging. (**A**) Response of astrocytes towards NIC [10 µM] after a pre-incubation with increasing concentrations of the non-selective nicotinic antagonist tubocurarine. (**B**) Response of astrocytes towards NIC [10 µM] after a pre-incubation with increasing concentrations of the α7 nAChR specific agonist AR-R-17779 (AR-R). (**C**) Response of astrocytes towards ACh [10 µM] after a pre-incubation with increasing concentrations of the mAChR specific antagonist atropin. (**D**) Response of astrocytes towards Ach [10 µM] after a pre-incubation with increasing concentrations of the nAChR antagonist tubocurarine. (**E**) Response of astrocytes towards a fixed concentration of ACh [10 µM] after a pre-incubation with varying concentrations of the nAChR agonist NIC. (**F**) Response of astrocytes towards ACh [10 µM] after a pre-incubation with increasing concentrations of the mAChR specific antagonist atropin and the nAChR antagonist tubocurarine. (**G**) Expression of astrocyte specific markers (GFAP, S100ß, or vimentin) was investigated by immunocytochemistry after chronic NIC exposure for 7 days. Representative images of cultures treated in three different ways are shown. Nuclei were stained with H-33342, which is indicated in blue pseudocolour in the images. Colours of immunostains are indicated for control (Ctrl.) cultures. Data is presented as % reactive cells normalized to the positive control. All data are mean ± SEM of biological triplicates; each biological replicate included three technical replicates. The 90% confidence intervals are indicated by dotted lines and grey background shading. Differences were tested for significance by two-way ANOVA, followed by Dunnett’s multiple comparison post hoc test for comparison between the treatments with the evoked rate of responders without pre-treatment of the stimulus. The Gaussian distribution of the residuals was tested with four tests (Anderson–Darling, D’Agostino–Pearson omnibus, Shapiro–Wilk, and Kolmogorvo–Smirnov). The results can the found in Supplementary Materials in the repository https://zenodo.org/records/20840279 (accessed on 25 June 2026).

In order to ascertain specificity of the nAChR responses, we profiled astrocytic responses to several established pharmacological tool compounds. In initial experiments, the non-selective nicotinic antagonist tubocurarine (Tubo) was used to inhibit the activation of the receptor [115,116,155]. Tubo blocked the response evoked by NIC [10 µM] at ≥0.1 µM, and it completely blocked any NIC signal at the HTC of 100 µM (Figure 3A). Secondly, we examined nAChR desensitization, an important feature of this receptor class [115,116]. Cells were exposed to the α7 nAChR subtype specific agonist AR-R 17,779 (AR-R) [115,116,156]. After 60 min, the Ca^2+^ signalling triggered by NIC [10 µM] was assessed. AR-R evoked a concentration-dependent desensitization of the nAChR at concentrations ≥ 1 µM. At concentrations above 10 µM, the response to NIC was completely abolished (Figure 3B).

After providing evidence for a specific reaction of the nAChR towards NIC by inhibition and desensitization approaches, we followed up on the response evoked by ACh in astrocytes. To investigate the contribution of mAChR to ACh signalling, the mAChR specific antagonist atropine was used. Concentrations of >10 µM decreased the response to ACh [10 µM] by 50% (Figure 3C). This incomplete inhibition indicates that non-muscarinic AChRs contribute to the ACh response. To inhibit the contribution of nAChR, astrocytes were pre-treated with increasing concentrations of Tubo. Similar to the effect observed by the pre-treatment with atropin, Tubo decreased the number of responders by approximately 50% at the highest tested concentration (100 µM) (Figure 3D). In a next step, astrocytes were pre-treated with a mixture of atropin and tubocurarine, followed by a stimulation with ACh [10 µM]. The combined inhibition of nAChR and mAChR inhibited the astrocytic response to ACh completely (Figure 3E).

To further characterize features of cholinergic signalling in our test system, we investigated the NIC-induced desensitization of the nAChR towards its physiological ligand, ACh. When astrocytes were pre-treated with NIC before stimulation with ACh, the Ca^2+^ signalling was attenuated. NIC concentrations as low as 0.1 µM showed an effect. At NIC concentrations > 1 µM, the number of responders was reduced by 75% (Figure 3F). This indicates that the acute exposure of astrocytes by NIC can lead to an altered reactivity of the cell population towards a physiological stimulus like ACh. This is in concordance with data from LUHMES cells, where it was shown that NIC and NeoNics can induce nAChR desensitization towards the physiological ligand ACh [115,116].

To investigate a potential cross-desensitisation to other receptor families, we explored the glutamate receptor family. Pre-treatment of astrocytes with increasing concentrations of NIC revealed no cross-desensitization for the stimulation of astrocytes with glutamate [100 µM] (Figure S3).

In a final approach, we explored whether a long-term (7-day) exposure to NIC may severely damage astrocytes. Gross morphological assessment did not reveal visible changes or cell losses. To provide a higher level of detail, astrocytic cultures were fixed and stained for cell phenotype markers, like GFAP, S100ß, and the intermediate filament VIM. Treatment with NIC had no significant effect on the expression or distribution of these antigens (Figure 3G and Figure S2). This confirms the fact that astrocytic viability is not affected by prolonged exposure to NIC at relatively high (10 µM) concentrations. However, it cannot be excluded that there were subtle metabolic effects or that some functional properties were altered. This is of interest for future toxicological studies. In the present work, we continued to focus on relatively short-term consequences of chemical exposure.

### 2.4. Comparison of Astrocytes and Neurons Concerning the Response Modulation to Thiacloprid

After providing evidence for the specificity of the nAChR response in astrocytes, we explored its toxicological applicability: (i) we chose a relevant toxicant (THI) and its bioactive metabolite (DCT) for this exemplary study, and (ii) we compared astrocytic responses to those of LUHMES neurons differentiated for 9 days (Figure S4A). For optimal comparability, neuronal Ca^2+^ signalling was analysed using the same experimental setup and analytical pipelines as for astrocytes [146]. The threshold fluorescence change to distinguish responder and non-responder neurons was based on an established statistical procedure parameterized by data from LUHMES neuron cultures [147,148]. According to this setup, 98% of all cell bodies responded to KCl stimulation. A normalization of subsequent data to the number of responsive cells was therefore obsolete (Figure S4B–H).

**Figure 4:**
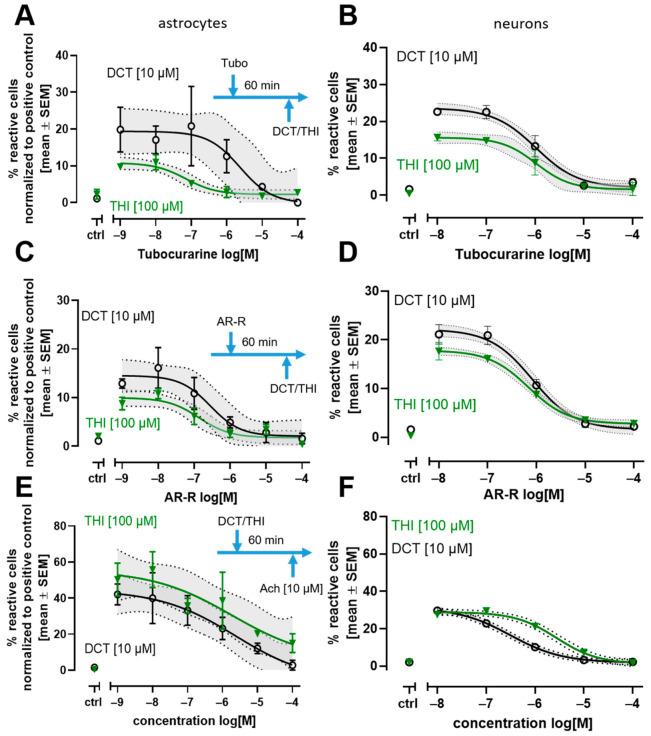
Modulation of the response to thiacloprid/descyanothiacloprid in astrocytes vs. neurons. Astrocytes (**A**,**C**,**E**) and neurons (**B**,**D**,**F**) were used in similarly designed experiments to assess Ca^2+^ responses. Astrocytes were used as in Figure 2. Dopaminergic human neurons (LUHMES) were differentiated in 96-well plates for 7 days. Then, they were loaded with a Ca^2+^ indicator dye and assayed, similar to the process described for astrocytes (Figure 1E). In all experiments, cells were stimulated with the neonicotinoid pesticide thiacloprid (THI) and its metabolite descyanothiacloprid (DCT). (**A**,**B**) Concentration–response of the nAChR antagonist tubocurarine in cells stimulated with THI or DCT. (**C**,**D**) Concentration–response of the α7 nAChR specific agonist AR-R in cells stimulated with THI or DCT. (**E**,**F**) De-sensitization of the ACh-triggered Ca^2+^-response by pre-treatment of cells with THI or DCT. Data is presented as % reactive cells normalized to the positive control for astrocytes and as % reactive cells for LUHMES cells. All data are mean ± SEM of biological triplicates; each biological replicate included three technical replicates. The 90% confidence intervals are indicated by dotted lines and grey background shading. Differences were tested for significance by two-way ANOVA, followed by Dunnett’s multiple comparison post hoc test for comparison between treatments with the evoked rate of responders without pre-treatment of the stimulus. The Gaussian distribution of the residuals was tested with four tests (Anderson–Darling, D’Agostino–Pearson omnibus, Shapiro–Wilk, and Kolmogorvo–Smirnov). The results can the found in Supplementary Materials in the repository https://zenodo.org/records/20840279 (accessed on 25 June 2026).

In the first set of comparative experiments, cells were pre-treated with Tubo and then stimulated by either THI or DCT. Tubo completely abolished the response to THI (BMC_10_ 0.07 µM and DCT (BMC_10_ 0.26 µM (C.I. 0.05–1.32 µM) in astrocytes (Figure 4A). Essentially similar data were obtained for neurons (THI (BMC_10_ 0.15 µM (C.I. 0.03–0.74 µM), DCT (BMC_10_ 0.19 µM (C.I. 0.02–1.74 µM)) (Figure 4B). In both test systems the inhibition by Tubo started at a BMC_10_ of 0.07–0.26 µM and reached a maximal effect at 10 µM.

The second comparison addressed nAChR desensitization: sstrocytes and LUHMES neurons were pre-treated with the α7 nAChR-specific agonist AR-R, followed by a stimulation with either THI or DCT after 60 min. In astrocytes AR-R, pre-treatment lead to a receptor desensitization at ≥0.02 µM for both THI (BMC_10_ 0.08 µM (C.I. 1.26 × 10^−73^–5.01 × 10^70^ µM) and DCT (BMC_10_ 0.02 µM (C.I. 0.001–0.48 µM) stimulation, and the triggered response was completely abolished at 10–100 µM (Figure 4C). In LUHMES neurons, essentially similar data were observed (THI (BMC_10_ 0.08 µM (C.I. 0.005–1.15 µM), DCT (BMC_10_ 0.14 µM (C.I. 0.02–0.91 µM) (Figure 4D).

Finally, we investigated the THI- or DCT-induced desensitization of the nAChR towards its physiological ligand ACh. Astrocytes and LUHMES neurons were pre-treated for 60 min with increasing concentrations of THI or DCT, and stimulated afterwards with ACh [10 µM]. In astrocytes, the NeoNics (THI (BMC_10_ 0.015 µM (C.I. 0.0002–1.20 µM), DCT (BMC_10_ 0.035 µM (C.I. 0.013–0.087 µM) induced a receptor desensitization at ≥0.035 µM, with DCT completely abolishing the response of ACh at the HTC of 100 µM (Figure 4E). In LUHMES neurons (THI (BMC_10_ 0.34 µM (C.I. 0.11–1.00 µM), DCT (BMC_10_ 0.03 µM (C.I. 0.007–0.11 µM), DCT induced a receptor desensitization at ≥0.03 µM, completely abolishing the triggered response by ACh at ≥10 µM (Figure 4F). Slight differences in the sensitivity of the model systems may be explained by a stronger contribution of mAChR activation by ACh in astrocytes.

### 2.5. Contribution of Voltage-Dependent Na^+^ and Ca^2+^ Channels to the Astrocytic and Neuronal Response to THI/DCT

**Figure 5:**
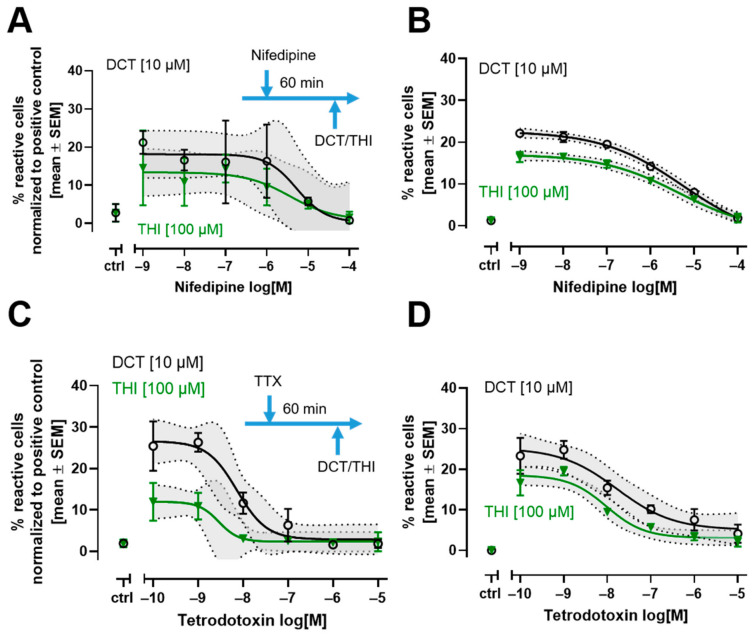
Contribution of voltage-dependent Na^+^ and Ca2^+^ channels to the astrocytic and neuronal response to thiacloprid/descyanothiacloprid. Astrocytes (**A**,**C**) and neurons (**B**,**D**) were used in similarly designed experiments to assess Ca^2+^-responses. Astrocytes were used as in Figure 2. Dopaminergic human neurons (LUHMES) were differentiated in 96-well plates for 7 days. Then, they were loaded with a Ca^2+^ indicator dye and assayed in a process similar to that described for astrocytes (Figure 1E). In all experiments, cells were stimulated with the neonicotinoid pesticide thiacloprid (THI) and its metabolite descyanothiacloprid (DCT). (**A**,**B**) Concentration–response of the L-type specific VDCC antagonist nifedipine in cells stimulated with THI or DCT. (**C**,**D**) Concentration–response of the Na_v_ antagonist tetrodotoxin (TTX) in cells stimulated with THI or DCT. Data is presented as % reactive cells normalized to the positive control for astrocytes and as % reactive cells for LUHMES cells. All data are mean ± SEM of biological triplicates; each biological replicate included three technical replicates. The 90% confidence intervals are indicated by dotted lines and grey background shading. Differences were tested for significance by two-way ANOVA, followed by Dunnett’s multiple comparison post hoc test for comparison between treatments with the evoked rate of responders without pre-treatment of the stimulus. The Gaussian distribution of the residuals was tested with four tests (Anderson–Darling, D’Agostino–Pearson omnibus, Shapiro–Wilk, and Kolmogorvo–Smirnov). The results can the found in Supplementary Materials in the repository https://zenodo.org/records/20840279 (accessed on 25 June 2026).

VDCCs are assumed to mediate the major component of Ca^2+^ signalling triggered by nAChR agonists on neurons [157], To test the involvement of VDCC in nicotinic responses of astrocytes, we investigated the inhibitory effect of nifedipine (Nif), an established pharmacological inhibitor of L-type voltage-dependent calcium channels (VDCCs) [158,159]. It decreased the response to THI (BMC_10_ 0.07 µM (C.I. 0.0009–5.01 µM) or DCT (BMC_10_ 0.5 µM (C.I. 0.02–10.96 µM) at ≥1 µM (Figure 5A). In LUHMES neurons, Nif was more potent and attenuated the response to THI (BMC_10_ 0.08 µM (C.I. 0.04–0.18 µM) or DCT (BMC_10_ 0.07 µM (C.I. 0.04–0.14 µM) at ≥0.1 µM (Figure 5B). This may be due to differential expression of various VDCC subtypes [110,160,161]. A large panel of tool compounds, together with genetic approaches, would be required for a detailed characterization.

While nAChR are ligand-gated Na^+^-channels, it is not clear whether the Na^+^ current from nAChR is sufficient for astrocytic depolarization and the subsequent Ca^2+^ influx through VDCC. To investigate the involvement of VGSCs in the reinforcement of the membrane depolarization by nAChR activation, we used tetrodotoxin (TTX) as a pharmacological inhibitor [162,163]. Astrocytes and LUHMES neurons were pre-treated for 60 min with increasing concentrations of TTX, followed by stimulation with either THI or DCT. In astrocytes, TTX attenuated the signal by THI (BMC_10_ 0.001 µM (C.I. 0.0003–0.003 µM) or DCT (BMC_10_ 0.0008 µM (C.I. 7.94 × 10^−5^–0.008 µM) at concentrations ≥ 0.0001 µM, completely abolishing the response at concentrations ≥ 1 µM (Figure 5C). In LUHMES neurons, similar effects were observed (THI (BMC_10_ 0.007 µM, DCT (BMC_10_ 0.003 µM (Figure 5D). Thus, astrocytes require at least two types of voltage-gated ion channels (VDCC and VGSC) for the reinforcement of toxicant-induced Ca^2+^ signalling.

### 2.6. Independent Control/Regulation of Nicotinic Ca^2+^ Responses and Inflammatory Responses in Astrocytes

After this initial profiling of the Ca^2+^ response of an exemplary nicotinic toxicant (THI) in human astrocytes, we asked whether there is any crosstalk between nicotinic signalling and inflammatory responses. This investigation was triggered by a wealth of literature [164,165,166,167,168] on the attenuation of inflammation by α7 nAChRs. The NFκB signalling pathway is a key regulator of inflammation in astrocytes. It was shown that nAChR activation can alter the NFκB signalling pathway in mouse astrocytes [169]. Therefore, we were interested in potential interactions of nicotinic signalling and NFκB.

**Figure 6:**
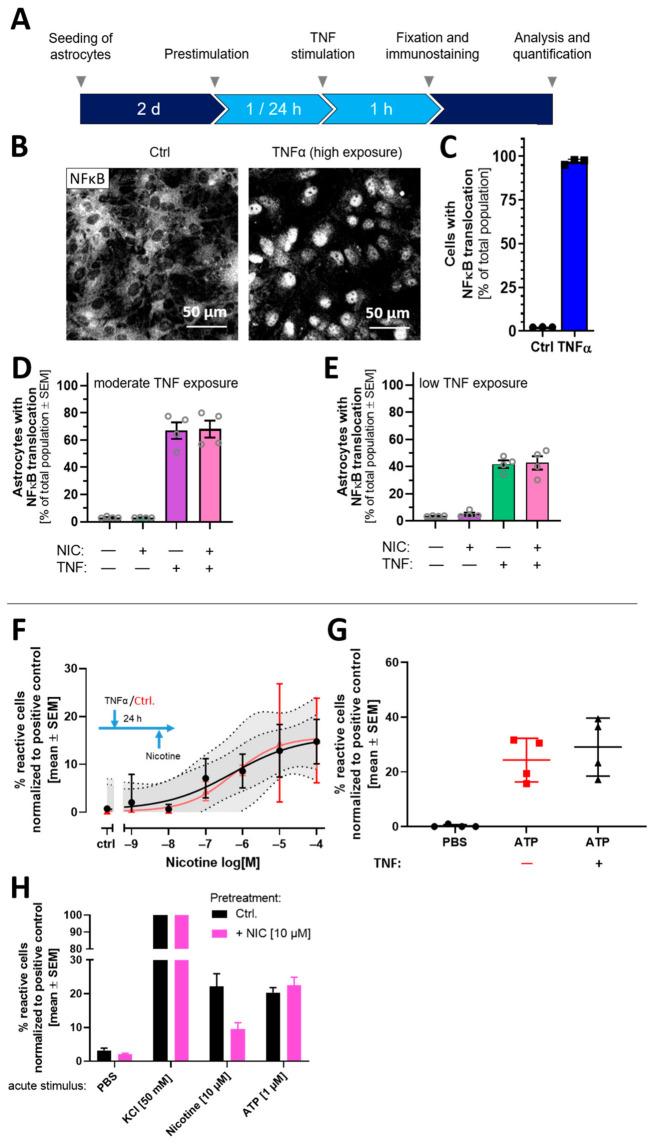
Independent control/regulation of nicotinic Ca^2+^ responses and inflammatory responses in astrocytes. Astrocytes (iPSC-derived) were cultured and used as in Figure 2, but experiments involved a pre-incubation step to clarify whether nicotine affected inflammatory TNFα responses or TNFα affected nicotinic Ca^2+^ responses. (**A**) Experimental set-up for the NFκB translocation assay, and its potential modulation. Astrocytes were pre-stimulated with nicotine for either 1 or 24 h, followed by exposure to TNFα [50/100 pg/mL] for 1 h. Cells were fixed and stained for NFκB (p65 subunit). The localization of NFκB (cytosol vs. nucleus) was identified and quantified automatically, and the number of cells with nuclear NFκB translocation was quantified. (**B**) Exemplary images of astrocytic NFκB distribution. Unstimulated astrocytes (Ctrl) had virtually all NFκB stain in their cytosol. The position of the nuclei is obvious from oval black holes. The TNFα stimulated astrocytes had virtually no cytosolic NFκB signal, while the nuclear area stained intensely. (**C**) Quantification of the fraction of cells with NFκB translocation. (**D**) Quantification of NFκB translocation after treatment with NIC [100 µM] for 1 h followed by stimulation with TNFα [100 pg/mL]. (**E**) Quantification of NFκB translocation after treatment with NIC [100 µM] for 24 h, followed by stimulation with TNFα [50 pg/mL]. (**F**,**G**) In these experiments, cells were pre-treated with TNFα [10 ng/mL] and then stimulated to assess their Ca2+ response. (**F**) Concentration–response curve of nicotine after 24 h pre-treatment with or without TNFα [10 ng/mL]. (**G**) Ca^2+^-response to ATP [10 µM] after 24 h pre-treatment with or without TNFα [10 ng/mL]. (**H**) Functional nAChR and purinergic receptor responses after chronic (7-day) pre-treatment with nicotine (NIC [10 µM]). Data is presented as % of astrocytes with NFκB translocation, or as % reactive cells normalized to the positive control for astrocytes. In (**A**–**E**) the data consists of four biological replicates with each four technical replicates, while (**F**–**H**) consist of three biological replicates with each three technical replicates. All data are mean ± SEM. The 90% confidence intervals are indicated by dotted lines and grey background shading. Differences were tested for significance by two-way ANOVA, followed by Sidak’s post hoc test for comparison between treatments. Comparison between two different groups in (**G**,**H**) were performed by a one-way ANOVA with a Tukey post hoc test for multiple comparison. The Gaussian distribution of the residuals was tested with four tests (Anderson–Darling, D’Agostino–Pearson omnibus, Shapiro–Wilk, and Kolmogorvo–Smirnov). The results can the found in Supplementary Materials in the repository https://zenodo.org/records/20840279 (accessed on 25 June 2026).

We used a previously established experimental set-up [54], in which NFκB translocation was triggered by TNFα (Figure 6A). Control experiments showed that the NFκB signal is mainly located in the cytoplasm of untreated astrocytes, while 10 ng/mL TNFα triggered a virtually complete translocation of the transcription factor to the nucleus (Figure 6B,C and Figure S7).

To investigate a modulatory role of nAChR activation on NFκB signalling in astrocytes, cells were pre-treated with NIC for 1 h before stimulation by TNFα. Nevertheless, the pre-treatment with NIC had no influence on NFκB translocation at all (Figure 6D). A prolonged exposure to NIC (for 24 h) also had no modulatory effect on NFκB translocation. Using a very low TNFα concentration (to increase the sensitivity of the modulation assessment) did also not reveal any effects of NIC (Figure 6E). In summary, NIC pre-treatment of human astrocytes did not alter the NFκB response triggered by TNFα. The effects earlier described for murine astrocytes [169] were thus not observed in our human test system.

In a next step, we wanted to clarify a potentially reverse interaction, i.e., whether pro-inflammatory stimulation of astrocytes by TNFα changed the function of nAChR receptors: astrocytes were strongly stimulated with TNFα [10 ng/mL] for 24 h, before stimulation with increasing concentrations of NIC. Untreated (BMC_10_ 35.48 µM (C.I. 11.48–109.65µM) and pre-treated (BMC_10_ 1.41 µM (C.I. 0.004–562 µM) astrocytes showed a similar increase in their response (Figure 6F). To check another ionotropic receptor, similar experiments were performed for stimulation by ATP [1 µM] instead of NIC. Here also, the inflammatory stimulation with TNFα did not have an effect on the responsiveness of astrocytes (Figure 6G).

In a final step, we explored whether long-term exposure (7 days) to NIC alters functional receptor expression of nAChR or purinergic receptors in astrocytes. Functional assessment revealed a desensitization of the nAChR after long-term exposure towards NIC [10 µM]. No change in the response of purinergic receptors (triggered by ATP) was observed in astrocytes pre-exposed to NIC. Thus, the nicotinic desensitization was not accompanied by a purinergic cross-desensitization.

In summary, we did not observe any connection between inflammatory responses and nicotinic Ca^2+^ signalling.

A limitation of the cell system is clearly that only a subpopulation (≤25%) of astrocytes is affected by nicotinic Ca^2+^ signalling. Thus, subtle effects might have been missed. In future studies, this may be explored by the addition of more inflammatory endpoints and the focus on the single-cell level. The objective of the current study was the characterisation of the effect of nicotinic toxicants, rather than nicotinic signalling in particular. Therefore, we continued by demonstrating the applicability of the astrocytes as a new test system to less-characterized compounds.

### 2.7. Selection of Cycloxaprid and Flupyradifurone for a Case Study on Astrocyte-Based Neurotoxicity Testing

The development of resistance in pests leads to the need for newly developed pesticides [170]. Two candidates that emerged in the last decade are cycloxaprid (CYC), a neonicotinoid-like compound that showed a good efficacy against sucking pests in populations that showed neonicotinoid resistance [171,172], and flupyradifurone (FPF), a butenolide-like compound which has a broader applicability and is listed as a tool for resistance management [173,174]. Both are predicted to bind to the nAChR [149,172,173], and it was shown in rodents that they may alter neural signalling [174]. However, so far, human data is scarce on these compounds.

In a first step, it was confirmed that this subset of neonicotinoid compounds was tolerated by astrocytes and neurons. An assessment of the neurite outgrowth and of the viability in mature and immature LUHMES neurons was performed [175]. None of the compounds showed cytotoxicity or neurite-specific effect (Figure S5). Further, we tested for cytotoxicity in astrocytes by propidium iodide (PI) staining and ATP measurements. None of the compounds showed an effect on the viability or ATP content at concentrations up to 100 µM (Figure S6).

In a second step, we addressed potential binding to the nAChR, based on docking studies and binding energy calculations (more negative ‘MMGBSA dG Bind’ values predict higher affinities). Nine nAChR docking models were set up [176,177,178], and mean binding energies (MM-GBSA dG bind values) were calculated. The predicted binding affinity of FPF and CYC was within a similar range to that of other commercial NeoNics, such as imidacloprid.

**Figure 7:**
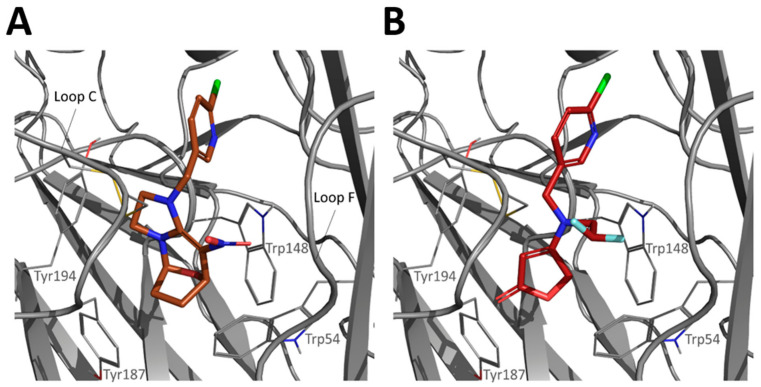
Docking of cycloxaprid (CYC) and flupyradifurone (FPF) to a α7 nAChR receptor model. A model of the homo-pentameric nAChR α7 (PDB-ID: 7koq) was established and used for in silico docking of potential ligands. Binding of cycloxaprid and flupyradifurone (brown and dark-red carbon atoms, respectively) are shown. Other colours are dark blue (for nitrogen), light blue (for fluorine), green (for chlorine), and orange-red (for oxygen) (**A**) CYC shows a conformation of the pyridine ring similar to nicotine, while pointing the nitroso moiety towards the loop C and loop F interface. (**B**) FPF shows a very similar orientation of the chlorine-substituted pyridine ring, but the difluoro group is directed towards the loop C/F interface, with the furan-one moiety facing the back of the binding site, where tyrosine residues (Tyr187, Tyr194) form the aromatic cage. The mean ‘MM-GBSA energy’ over nine nAChR α7 model variants was measured and resulted in the following binding energies [in kcal/mol]: FPF—38, CYC—34, Imidacloprid—36, DN-IMI—38, Descyanothiacloprid—37, Nicotine—34, and epibatidine—61.

Subsequently, we followed up on the binding affinity by comparing the binding mode (topology of the receptor) of CYC and FPF. Repeated model runs showed that CYC (Figure 7A) and FPF (Figure 7B) bound in a similar location, interacting with similar receptor parts as NIC. Moreover, both binding modes, the common- and inverted-binding orientation, which are described for NeoNics on mammalian nAChRs [139,179], were observed. The ‘common binding mode’ is characterized by alignment of the NeoNic pyridine rings with that of agonists like nicotine or epibatidine. The ‘inverted mode’ is reversed in its orientation. These data suggest that CYC and FPF indeed bind the human nAChR in a similar way as other agonistically acting NeoNics. Based on this, we decided to use our test methods for a more detailed functional characterization of the two compounds.

### 2.8. Characterization of Signalling Disturbances by Cycloxaprid and Flupyradifurone in iPSC-Derived Astrocytes and LUHMES Neurons

**Figure 8:**
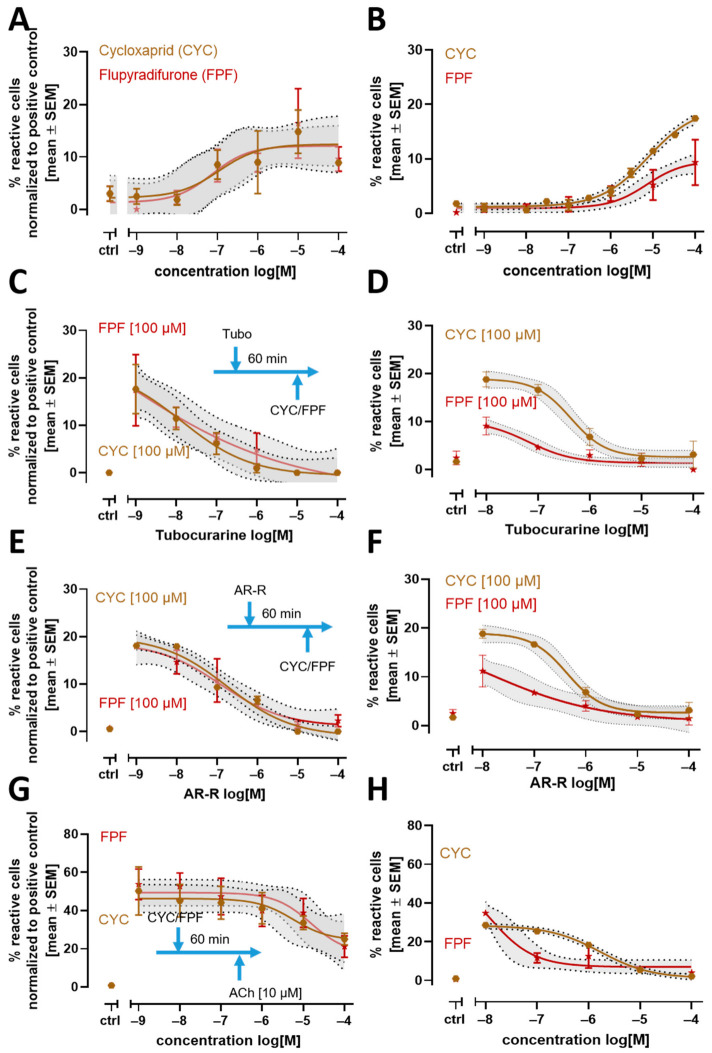
Basic characterization of a subgroup of neonicotinoid pesticides in hiPSC-derived astrocytes and neurons. Astrocytes (**A**,**C**,**E**) and neurons (**B**,**D**,**F**) were used in similarly designed experiments to assess Ca^2+^ responses. Astrocytes were used as in Figure 2. Dopaminergic human neurons (LUHMES) were differentiated in 96-well plates for 7 days. Then, they were loaded with a Ca^2+^ indicator dye and assayed, in a similar process as that as described for astrocytes (Figure 1E). In all experiments, cells were stimulated with the neonicotinoid pesticide cycloxaprid (CYC) or flupyradifurone (FPF). (**A**,**B**) Concentration–response of the neonicotinoid pesticides CYC and FPF. (**C**,**D**) Concentratio–response of the nAChR antagonist tubocurarine in cells stimulated with CYC or FPF. (**E**,**F**) Concentration–response of the α7 nAChR specific agonist AR-R in cells stimulated with CYC or FPF. (**G**,**H**) De-sensitization of the ACh-triggered Ca^2+^-response by pre-treatment of cells with CYC or FPF. Data is presented as % reactive cells normalized to the positive control for astrocytes and as % reactive cells for LUHMES cells. All data are mean ± SEM of biological triplicates; each biological replicate included three technical replicates. The 90% confidence intervals are indicated by dotted lines and grey background shading. Differences were tested for significance by two-way ANOVA, followed by Dunnett’s multiple comparison post hoc test for comparison between treatments with the evoked rate of responders without pre-treatment of the stimulus. The Gaussian distribution of the residuals was tested with four tests (Anderson–Darling, D’Agostino–Pearson omnibus, Shapiro–Wilk, and Kolmogorvo–Smirnov). The results can the found in Supplementary Materials in the repository https://zenodo.org/records/20840279 (accessed on 25 June 2026).

The pesticides CYC and FPF were used for a comparative study of astrocytes and human neurons (LUHMES), to generate new toxicological data. Initially, cells were treated with increasing concentrations of CYC and FPF. In astrocytes, CYC (BMC_10_ 0.54 µM (C.I. 1.58 × 10^−5^–16,218 µM)) triggered a response of approximately 15% responders at concentrations of ≥10 µM. FPF (BMC_10_ 0.35 µM (C.I. 0.0001–1148 µM)) evoked a comparably strong response (Figure 8A). The responder rate in LUHMES neurons was similar (CYC (BMC_10_ 6.61 µM (C.I. 2.45–17.78 µM)), FPF (BMC_10_ not reached)) to the one in astrocytes (8–20% range). On first view, astrocytes appeared to be more sensitive than LUHMES (based on curve shapes). However, the astrocyte response also had a higher variability. Due to this statistically significant effect, sizes were reached in LUHMES at the same or lower concentrations. Both systems identified signalling effects of the toxicants in the 10 µM range, and were thus providing largely similar information (Figure 8B).

To test specificity for the nAChR, cells were pre-treated with Tubo. It inhibited the response triggered by either CYC [100 µM] (astrocyte BMC_10_ 0.002 µM (C.I. 0.0005–0.006 µM)), LUHMES BMC_10_ 0.05 µM (C.I. 0.003–1.17 µM)) or FPF [100 µM] (astrocyte BMC_10_ 0.001 µM (C.I. 0.0001–0.01 µM)), LUHMES BMC_10_ 0.004 µM (C.I. 5.01 × 10^−5^–0.36 µM)) to a large degree, at ≥1 µM (Figure 8C,D). Based on this, we conclude that both compounds disturb Ca^2+^ signalling via triggering of α7 nAChRs.

To confirm receptor specificity, desensitization experiments were performed. Cells were pre-treated for 60 min with the α7 nAChR specific agonist AR-R, followed by stimulation with either FPF or CYC. Desensitization by AR-R (≥0.01 µM) inhibited the responses to CYC (astrocyte BMC_10_ 0.009 µM (C.I. 0.0009–0.09 µM)), LUHMES BMC_10_ 0.05 µM (C.I. 0.003–1.17 µM)) or FPF (astrocyte BMC_10_ 0.003 µM (C.I. 0.0005–0.02 µM)), LUHMES BMC_10_ 0.007 µM (C.I. 0.0003–0.19 µM)), to a large degree (>90%) (Figure 8E,F).

We also asked whether the exposure to CYC or FPF would alter (desensitize) the reaction of astrocytes or LUHMES to physiological signalling by ACh. Astrocytes, pre-treated with CYC (BMC_10_ 0.06 µM (C.I. 0.012–0.31 µM)) or FPF (BMC_10_ 0.11 µM (C.I. 0.008–1.74 µM)), showed a desensitization of the nAChR at concentrations ≥ 0.1 µM. At the HTC, the ACh response was attenuated by approximately 50% (Figure 8F). In LUHMES neurons, pre-treatment with CYC (BMC_10_ 0.11 µM (C.I. 0.05–0.28 µM)) induced a desensitization at concentrations ≥ 0.1 µM. At the HTC of CYC the ACh response was completely blunted. FPF (BMC_10_ 0.03 µM (C.I. 6.31 × 10^−47^–1.58 × 10^43^ µM)) pre-treatment also completely inhibited the LUHMES response to ACh (Figure 8G). The more pronounced desensitization effects in neurons (100%) compared to astrocytes (50%) are well in line with our findings that astrocytes also express mAChR, which contributes to the overall Ca^2+^ response, but they are not desensitized by the toxicants. One cell-type difference may be that FPF or CYC showed essentially similar effects in astrocytes over a broad range of conditions, while in LUHMES cells CYC triggered a larger responder rate than FPF. The mechanistic rationale for this remains unclear, but it may be due to slight differences in the interaction with non-α7 nAChR subtypes, known to be expressed in LUHMES cells.

Overall, the data between the two test systems are in good concordance with each other, indicating a similar sensitivity towards the activation of nAChR by these pesticides. We followed this initial characterization by investigating the involvement of VDCCs and VGSCs.

**Figure 9:**
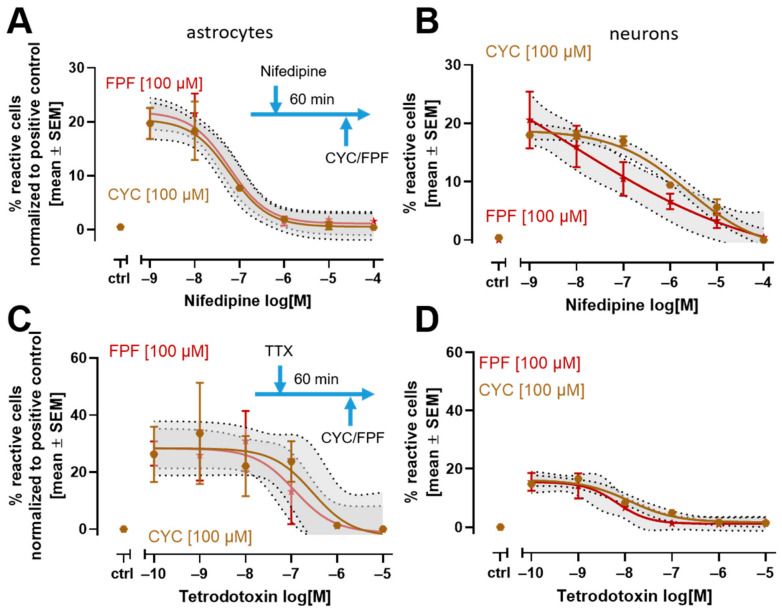
Signalling modulation of a subgroup of neonicotinoid- and butenolide-like pesticides in astrocytes and neurons. Astrocytes (**A**,**C**) and neurons (**B**,**D**) were used in similarly designed experiments to assess Ca^2+^-responses. Astrocytes were used as in Figure 2. Dopaminergic human neurons (LUHMES) were differentiated in 96-well plates for 7 days. Then, they were loaded with a Ca^2+^ indicator dye and assayed, in a similar process as that described for astrocytes (Figure 1E). In all experiments, cells were stimulated with the neonicotinoid pesticide cycloxaprid (CYC) or flupyradifurone (FPF). (**A**,**B**) Concentration–response of the L-type specific VDCC antagonist nifedipine in cells stimulated with CYC or FPF. (**C**,**D**) Concentration–response of the Na_v_ antagonist tetrodotoxin (TTX) in cells stimulated with CYC or FPF. Data is presented as % reactive cells normalized to the positive control for astrocytes and as % reactive cells for LUHMES cells. All data are mean ± SEM of biological triplicates; each biological replicate included three technical replicates. The 90% confidence intervals are indicated by dotted lines and grey background shading. Differences were tested for significance by two-way ANOVA, followed by Dunnett’s multiple comparison post hoc test for comparison between treatments with the evoked rate of responders without pre-treatment of the stimulus. The Gaussian distribution of the residuals was tested with four tests (Anderson–Darling, D’Agostino–Pearson omnibus, Shapiro–Wilk, and Kolmogorvo–Smirnov). The results can the found in Supplementary Materials in the repository https://zenodo.org/records/20840279 (accessed on 25 June 2026).

To conclude the characterization of the response evoked by CYC and FPF, we profiled the involvement of VDCCs and VGSCs in the response. Nif and TTX were used as tool compounds to block L-type VDCCs, and VGSCs. Nif pre-treatment in astrocytes completely blocked a response to CYC/FPF (CYC BMC_10_ 0.01 µM (C.I. 0.004–0.025 µM)) and FPF BMC_10_ 0.07 µM (C.I. 2 × 10^−36^–2.51 × 10^33^ µM)) at concentrations ≥ 10 µM (Figure 9A). In LUHMES neurons, Nif pre-treatment attenuated the evoked response by CYC (BMC_10_ 0.08 µM (C.I. 0.014–0.40 µM)) and FPF (BMC_10_ 0.002 µM (C.I. 0.0002–0.009 µM)) at concentrations ≥ 0.1 µM, completely abolishing a response at the HTC of 100 µM (Figure 9B). This data agrees well with previous findings on other NeoNics (THI, DCT, Figure 5A,B).

To inhibit the opening of VGSCs after membrane depolarization by nAChR activation, astrocytes and LUHMES neurons were pre-treated with increasing concentrations of TTX. In astrocytes (CYC BMC_10_ 0.056 µM (C.I. 0.004–0.76 µM)) and FPF BMC_10_ 0.079 µM (C.I. 1 × 10^−169^–1 × 10^167^ µM)), a complete inhibition was observed at concentrations ≥ 1 µM (Figure 9C). In LUHMES neurons, the effects were essentially similar (CYC BMC_10_ 0.0009 µM (C.I. 6.31 × 10^−5^–0.012 µM)), FPF BMC_10_ 0.001 µM (C.I. 0.0002–0.008 µM)) (Figure 9D). The data is in good concordance with the findings on THI and DCT (Figure 5C,D).

In summary, this case study showed how potential neurotoxicity of new compounds may be explored and compared in two different neural test systems representative of major central nervous-system cell types.

## 3. Discussion

In the present study, we explored human astrocytes from a single iPSC line, therefore not capturing inter-donor/inter-line variability, as a test system to study acute functional effects of toxicants on brain cell communication. After a broader characterization of signalling elements that may be assessed by measuring [Ca^2+^]_i_, we proceeded towards the characterization of neonicotinoids as an application case study. Initially, effects of compounds known to affect neurons were compared to astrocytes. Here, as in all subsequent study parts, data were obtained for relatively large concentration ranges to allow comparisons not only of efficacy, but also of potency. In a final step, data-poor compounds were de novo characterized in astrocytes, as well as neurons. Our data provide strong evidence that astrocytes need to be considered as target cells of a potential neonicotinoid-mediated neurotoxicity. On a more general level, our study suggests that iPSC-derived astrocytes may be used as a model system to study the interference of potential toxicants with cholinergic signalling. Of particular interest for the future may be the fact that both nAChR and mAChRs were functionally active. The data on nAChR interactions were in good concordance with data obtained in LUHMES neuron-like cells, where THI, DCT, IMI, and DN-IMI triggered a response, while thiamethoxam did not trigger a response [115,116,139]. The nAChR is mainly permeable to Na^+^ [157], while here the Ca^2+^ flux was used as the main endpoint. All measurements for nAChR activation were therefore measured in the presence of the positive allosteric modulator PNU to increase the opening time of the receptor to enable measurements of changes in [Ca^2+^]_i_. While this situation does not mimic the physiological condition, it was shown by patch clamp experiments in a previous study that the activation in the absence of PNU still leads to an influx of Na^+^ and therefore a change in the membrane potential [115,116].

Astrocytes are known to release several gliotransmitters in a Ca^2+^-dependent manner. The link between the inputs of information to an astrocyte (e.g., via nAChRs) to its output responses is likely to be encoded by the type and strength of Ca^2+^ signals [180]. A further modifying factor, not studied here, are Ca^2+^ oscillations within astrocytes or entire networks of coupled (by gap junctions) astrocytes [181,182,183]. We found here, by some initial experiments, that VGSCs and VDCCs strongly contributed to the changes in [Ca^2+^]_i_ triggered by older and newer NeoNics. Further studies may address further signal modifiers and more complex signal modalities.

Besides their signalling role [7,8,9,10,11,12,13], astrocytes provide structural and metabolic support to other brain cells [2,3,4,5,6]. As no obvious morphological changes were observed, our study did not clarify whether prolonged exposure of astrocytes to NIC triggers long-term or permanent changes in the brain. The fast desensitization of nAChRs after activation [115,116] may contribute to relative resilience of astrocytes. Functional testing (altered glutamate signalling after prolonged NIC exposure) also did not indicate a change of astrocytes due to 7-day exposure to NIC.

Astrocytes can switch between supporting roles in the CNS and a reactive state during injury or inflammation. The reactive state can be either protective or harmful, depending on the trigger or disease stage [184,185,186]. It has been shown previously that the activation of the α7 nAChR can dampen the NFκB response in mouse astrocytes [169]. This might be important, since activated glial cells may contribute to the loss of dopaminergic neurons in diseases like Parkinson’s Disease (PD) [187]. Here, we did not observe any changes in inflammatory signalling upon the pre-stimulation with NIC for up to 24 h.

The concentration of NIC used here was in the range detected (0.04–0.36 µM) in heavy smokers [188]. In the foetus, even higher concentrations (~15% higher) may be reached, due to NIC accumulation during pregnancy [115,116,189,190]. Early life-stage exposure to NIC has been documented to cause long-lasting behavioural effects [79,191,192,193,194]. While developmental neurotoxicity (DNT) of nicotine is well-established [195], the situation for other nAChR agonists is complex. On the one hand, two OECD guideline studies (TG426) have been performed for IMI, with negative results in rats [196]. On the other hand, mechanistic studies suggested increased neuroinflammation, oxidative stress, persistently affected cholinergic signalling, and progressively suppressed neurogenesis in various models [30,197,198,199,200,201,202]. Further, IMI may form metabolites (e.g., DN-IMI) that permeate the mammalian blood-barrier and are more potent than the parent compound [203]. This situation illustrates the need for a panel of human-relevant models that allow measurements of subtle effects or functional disturbances.

Our study presents one such new test system for the identification and mechanistic study of neurotoxicants. While comparative studies showed a good reproducibility of test outcomes of neurons and astrocytes, the additional availability of the astrocyte model increases the biological coverage of possible targets and adverse effects in the central nervous system. Future studies may use the fact that LUHMES cells and astrocytes are compatible with co-culture conditions [53], and may thus be used in 2D- or 3D-culture formats.

## 4. Materials and Methods

### 4.1. Stem Cell Culture

The induced pluripotent stem cells (iPSCs) (Si28, EPITHELIAL-1, #IPSC0028; Sigma-Aldrich, Traufkirchen, Germany) were cultured in mTesR1 medium (StemCell Technologies, Vancouver, BC, Canada) on Matrigel-coated (Corning, Somerville, MA, USA) tissue culture dishes (60 mm, Sarstedt, Nümbrecht, Germany) and passaged at 80–90% confluency using EDTA. Cells were incubated at 37 °C and 5% CO_2_, and the medium was changed daily.

### 4.2. Differentiation and Culture of iPSC-Derived Astrocytes

Astrocytes were produced in-house, according to the protocol described earlier [54,145] and used for experiments between days 70 and 130 of differentiation. For cryopreservation, cells were frozen in astrocyte differentiation medium composed of DMEM/F12 supplemented with 1× N2 and 1× B27 (both from Gibco, Thermo Fisher Scientific, Waltham, MA, USA), 2 mM L-glutamine (Sigma-Aldrich, Taufkirchen, Germany), 1% fetal bovine serum (FBS; PAA Laboratories, Cölbe, Germany), and 10% dimethyl sulfoxide (DMSO; Merck, Darmstadt, Germany).

For experiments, frozen astrocytes were thawed, and 10 mL DMEM/F12 was added, followed by a centrifugation step at 500× *g* for 4 min. The cell pellet was resuspended in astrocyte differentiation medium and plated at a density of 60,000 cells/cm^2^ on coated plates. Plates were coated with Matrigel (1:40 dilution in DMEM/F12) for 30 min at 37 °C and 5% CO_2_, and excess coating solution was removed immediately before seeding. Astrocytes were passaged weekly using Accutase (5–8 min, 37 °C, 5% CO_2_). Detached cells were washed with DMEM/F12, centrifuged at 500× *g* for 4 min, resuspended in astrocyte differentiation medium, and re-seeded as described above.

### 4.3. LUHMES Cell Culture

LUHMES cells (ATCC CRL-2927^™)^ were cultured as described above [204,205]. The 96-well plates (Sarstedt, Nümbrecht, Germany) were coated with 100 µL poly-L-ornithine (PLO) (50 µg/mL) (Sigma-Aldrich, Merck, Darmstadt, Germany) in phosphate-buffered saline (PBS) (w/o Ca^2+^ and Mg^2+^). After one night at 37 °C, the solution was discarded. Plates were washed three time with PBS. Fibronectin (1 µg/mL) (Sigma-Aldrich, Merck, Darmstadt, Germany) and laminin (1 µg/mL) (Sigma-Aldrich, Merck, Darmstadt, Germany) in PBS were added to each well and they were incubated overnight at 37 °C. The solution was discarded directly before the use of the multi-well plates. LUHMES cells were maintained in T75 flasks (Sarstedt, Nümbrecht, Germany) in proliferation medium (PM). PM consisted of advanced DMEM/F12 medium (Gibco, Rockville, MD, USA) supplemented with N2 supplement (1×) (Invitrogen, Karlsruhe, Germany), glutamine (2 mM) (Gibco, Rockville, MD, USA), and recombinant basic fibroblast growth factor (40 ng/mL) (bFGF, 4114-TC, R&D Systems, Minneapolis, MN, USA). Cells were split every two days, when reaching 80% confluence. Before splitting, the cells were washed once with PBS, then detached with 0.05% trypsin (Sigma-Aldrich, Merck, Darmstadt, Germany), collected in non-supplemented medium centrifuged at 340× *g* for 4 min, re-suspended in medium and counted with a hemocytometer (Neubauer) chamber. The cells were then seeded in a T75 flask with 15 mL of PM or differentiation medium (DM), respectively (day-1, pre-differentiation). DM consisted of advanced DMEM/F12 supplemented with glutamine (2 mM) (Gibco, Rockville, MD, USA), cAMP (1 mM) (Sigma-Aldrich, Merck, Darmstadt, Germany), tetracycline (2.25 µM) (Sigma-Aldrich, Merck, Darmstadt, Germany), and glial cell-derived neurotrophic factor (2 ng/mL) (GDNF, 212-GD (50 µg), Bio-Techne, Minneapolis, MN, USA). Three million cells were used for maintenance, three million were used for pre-differentiation, and nine million were used for differentiation. One day after seeding (d0), the PM was exchanged for the DM. The PM was aspirated, the flask was washed with 10 mL PBS, and, afterward, 15 mL of DM was added. LUHMES cells were then cultivated for two days in the T75 flask and then seeded into 96-well plates at a density of 60,000 cells/well (207,000 cells/cm^2^) for experiments on day 9 (d9). On days 4, 6, and 8, 50% of the medium volume was renewed. Calcium signalling experiments were performed on d9.

### 4.4. Ca^2+^ Imaging

Ca^2+^ imaging was performed using a Cellomics Arrayscan VTI HCS Reader (Thermo Fisher Scientific, Waltham, MA, USA) equipped with an automated pipettor and an incubation chamber. Measurements were recorded at nominal 37 °C and 5% CO_2_. The Cellomics Arrayscan allows for the recording of indirect changes in [Ca^2+^]_i_ via a Ca^2+^-sensitive fluorescent dye. The Cellomics Arrayscan VTI HCS Reader acquires the fluorescence signal of one well at a time. The integrated pipet unit allows a controlled compound administration into one well. Cells were imaged continuously, as fast as possible, for 45 s at approximately 2 frames per second (fps). Compounds were administered automatically after 10 s of baseline recording. The images were exported as 16-bit.tiff image files and analysed in an adapted CaFFEE software version 2 [146]. Human iPSC-derived astrocytes were seeded for 2 days in a 96-well plate at a density of 14,500 cells per well (45,312 cells/cm^2^) in differentiation medium consisting of SILACTM advanced DMEM/F-12 FLEX supplemented with glutamine (2 mM) (Gibco, Rockville, MD, USA), Arginine (500 nM), Lysine (500 nM), Glucose (18 mM), cAMP (1 mM) (Sigma-Aldrich, Merck, Darmstadt, Germany), tetracycline (2.25 µM) (Sigma-Aldrich, Merck, Darmstadt, Germany), and glial cell-derived neurotrophic factor (2 ng/mL) (GDNF, 212-GD (50 µg), Bio-Techne, Minneapolis, MN, USA). LUHMES cells were differentiated for 2 days in a T75 flask. On differentiation day 2, cells were seeded into 96-well plates at a density of 60,000 cells/well (210,000 cells/cm^2^) and cultivated until differentiation day 9. Cells were incubated with Cal-520AM (AAT Bioquest) for 1 h at a concentration of 5 µM at 37 °C. The fluorescence Ca^2+^ indicator solution contained PNU-120596 (10 µM) (Sigma-Aldrich, Merck, Darmstadt, Germany); an allosteric modulator for the α7 nicotinic receptor, probenecid (1.54 mg/mL) (Thermo Fisher Scientific, Karlsruhe, Germany); an inhibitor of organic anion transporters located in the cell membrane; and PluronicTM-F127 (0.4%) (Thermo Fisher Scientific, Karlsruhe, Germany), a non-ionic tensid-polyol which helps with the dispersion of the dye.

### 4.5. Antagonist and Agonist Experiments

On the day of measurement, 75 µL of differentiation medium was replaced by 25 µL of a Cal-520 AM dye solution, containing PNU-120596, Probenecid and PluronicTM-F127. Additionally, the dye solution contained the antagonist or agonist at the respective concentration, which was incubated with the dye solution for 1 h before treatment. Fluorescence intensity was measured and data was analysed with CaFFEE software (V4.0).

### 4.6. Calcium Fluorescent Flash Evaluating Engine (CaFFEE)

CaFFEE enables the user to process and evaluate large quantities of image data. It automatically identifies individual cells in mixed, heterogeneous populations and evaluates their fluorescent signal. It enables the evaluation of the influence of a treatment on the [Ca^2+^]_i_ of hundreds of cells in a single well. The data can be exported in spreadsheet format. Moreover, the image data can be processed for an optimized visual representation of the time-lapsed image data, which can be explored by setting the parameters for semi-automated data processing [146]. The adapted CaFFEE identifies cells automatically by their morphological features of the somata. After the identification of a cell, CaFFEE defines each cell as a region of interest. The average fluorescent intensity of these pixels is measured over a series of pictures, thereby obtaining time-dependent fluorescent values for each cell. This information is then converted into curves from which different parameters can be obtained. With CaFFEE, the timepoint of peak fluorescence can be obtained. Fluorescent data for baseline recording allowed for the automated assessment of the ground state (F0) and peak timepoint (F1) for every individual cell. The difference between base level and peak level in fluorescence, ΔF = F1 − F0, was used for further data analysis. The noise-level-based threshold, mean ΔF + 3 × SDΔF, defining a reactive or non-reactive cell, was determined as follows: the mean ΔF value was calculated from all wells that received a negative stimulus (differentiation medium). A cell was defined as reactive when ΔF stimulus > threshold or non-reactive when ΔF stimulus < threshold.

### 4.7. Immunostaining

Astrocytes were seeded on glass coverslips (Thermo Fisher Scientific, Waltham, MA, USA) or in pre-coated 96-well plates at a density of 40,000 cells/cm^2^ or on 8-well IBIDI plates at a density of 45,000 cells/cm^2^. Cells were fixed by replacing the culture medium with 10% neutral buffered formalin (Leica Biosystems Richmond, Inc., Richmond, IL, USA) in PBS and incubating for 10 min at room temperature. Following the fixation, cells were washed once with PBS and permeabilized with 0.6% Triton X-100 (Sigma-Aldrich, Taufkirchen, Germany) in PBS for 10 min at room temperature. Non-specific binding was blocked using blocking buffer (0.1% Triton X-100 and 5% FBS in PBS) for 1 h at room temperature. Cells were then incubated with the respective primary antibodies (Table S1) diluted in blocking buffer overnight, at 4 °C. After removal of unbound antibodies, cells were washed three times with DPBS and subsequently incubated with the corresponding secondary antibodies (Table S1) diluted in blocking buffer for 30–60 min at room temperature. Nuclei were counterstained with Hoechst-33342 (Merck, Darmstadt, Germany) during secondary antibody incubation.

After three washes with DPBS, cells in 96-well plates or 8-well IBIDI plates were stored in PBS at 4 °C, until imaging. Coverslips were mounted face down on glass slides using Aqua-Poly/Mount (Polysciences, Warrington, PA, USA), left to dry overnight at room temperature, and stored at 4 °C until further use. Imaging was performed using a Zeiss LSM 770 confocal microscope equipped with a 63×/1.4 Plan-Apochromat oil immersion objective (Zeiss, Oberkochen, Germany), and all images were processed in ImageJ (FIJI v1.54f).

### 4.8. NFκB Translocation

For the quantification of NFκB translocation, 96-well plates were pre-coated with poly-L-ornithine hydrobromide (43 µg/mL; Sigma-Aldrich, Traufkirchen, Germany) in Milli-Q H_2_O overnight, at 37 °C. After three washes with Milli-Q H_2_O, plates were coated with laminin and fibronectin (1 µg/mL each; both from Sigma-Aldrich, Taufkirchen, Germany) in Milli-Q H_2_O, and incubated overnight at 37 °C. The coating solution was aspirated immediately before seeding astrocytes at a density of 40,000 cells/cm^2^ in astrocyte differentiation medium. Experiments were performed two days after seeding. Astrocytes were treated with nicotine (Sigma-Aldrich, Steinheim, Germany) and/or TNFα (R&D Systems, Minneapolis, MN, USA), and incubated at 37 °C and 5% CO_2_ for either one or twenty-four hours. Cells were then fixed by replacing the culture medium with 10% neutral buffered formalin (Leica Biosystems Richmond, Inc., Richmond, IL, USA) and subsequently incubated for 10 min at room temperature. Fixation solution was removed, and cells were stored in DPBS at 4 °C until further processing. Immunofluorescence staining was carried out as described above, using an anti-NFκB p65 antibody (Table S1). Nuclei were counterstained with Hoechst-33342 (Merck, Darmstadt, Germany).

NFκB translocation was quantified using a Cellomics ArrayScan automated fluorescence microscope (Thermo Fisher Scientific, Waltham, MA, USA) equipped with the predefined “nuclear translocation” algorithm, as described previously [54]. In brief, nuclear outlines were defined based on the H-33342 staining. The mean pixel intensity of the NFκB signal within the nuclear region (3.3 µm = 5 pixels from the nuclear outline toward the centre) and in the cytoplasmic ring (width 2.6 µm = 4 pixels and 3.3 µm = 5 pixels from the nuclear outline toward the cell membrane) were determined. The ratio of the average nuclear-to-cytoplasmic NFκB signal intensities was calculated for each cell. Cells were classified as “activated” when this ratio exceeded the mean ratio of unstimulated reference cells by at least one standard deviation. The average intensity ratio of the reference wells was automatically obtained. For each well, 300 cells were analysed. Four independent astrocyte differentiations, with four technical replicate wells per differentiation were used for analysis.

### 4.9. In Silico Docking Studies

The protein structures of human nAChRs that were used for the docking studies were downloaded from the Protein Data Bank [206]. The following nAChR isoforms were used: α4β2 (PDB-IDs: 6cnj, 6cnk [176]), α3β4 (PDB-IDs: 6pv7, 6pv8 [177]), and α7 (PDB-IDs: 7kox, 7koq [178]). Since the structures of nAChR α3β4 and α7 contain a co-crystallised water molecule within the binding site [177,178], its effect upon binding was investigated by generating two variants: the presence or absence of the water molecule is indicated with the suffixes ws1 and ws2, respectively. Hence, a total of nine protein structures (6cnj, 6cnk, 6pv7-ws1, 6pv7-ws2, 6pv8-ws1, 6pv8-ws2, 7kox-ws1, 7kox-ws2, and 7koq) were used for the ensemble docking study. Protein and ligand preparation, as well as the pose selection protocol, are described in [139]. The MM-GBSA (molecular mechanics generalized Born Surface Area model) tool was used for rescoring of the representative binding modes by including the ligand and binding-site residues lining within 5 Å for minimisation [207]. MM-GBSA was applied with the VSGB2.1 implicit solvent model [208] and OPLS4 force field [209]. Pymol (Version 2.5, [210]) was used to generate the figures of the proposed binding modes.

### 4.10. Data Analysis

Data was analysed using the software CaFFEE, as described earlier. Further analysis was performed in Excel. Visualisation was done using GraphPad Prism 8. Each data point consists of three biological replicates with each of the three technical replicates. Each technical replicate represents one well in an experiment with several dozen of cells. For each concentration–response curve, a benchmark concentration 10 (BMC_10_) was calculated, using the BMCeasy program [211]. Upper and lower bounds of the BMC_10_ are given as C.I. lower bound–upper bound in the main text, and in detail in Table S2 in the Supplementary File.

### 4.11. Data Handling and Statistics

All data sets include at least three repeats. Information about exact replicate numbers can be taken from the data repository on Zenodo (doi: 10.5281/zenodo.20840279). Unless stated otherwise, error bars represent the mean ± SEM. A *p*-value of <0.05 was considered statistically significant. For comparison between different concentration–response curves, a two-way ANOVA with a Sidak post hoc test was performed. For testing for statistical significance within a concentration–response curve against the untreated control or against the non-pretreated stimulation, a two-way ANOVA with a Dunnett post hoc test was performed. Comparison between two different groups in Figure 6 was performed by a one-way ANOVA with a Tukey post hoc test for multiple comparison. For each statistical test, the Gaussian distribution of the residuals was tested with four tests (Anderson–Darling, D’Agostino–Pearson omnibus, Shapiro–Wilk, and Kolmogorvo–Smirnov). The 95% confidence intervals are used as the effect-size measure. All data presented in manuscript figures are available in Excel files, so that other displays or statistical approaches may be applied to them. A possible statistical evaluation for the tests used as described above is given in Supplementary Materials in the data repository on Zenodo, with the following doi: 10.5281/zenodo.20840279.

## 5. Conclusions

**Figure 10:**
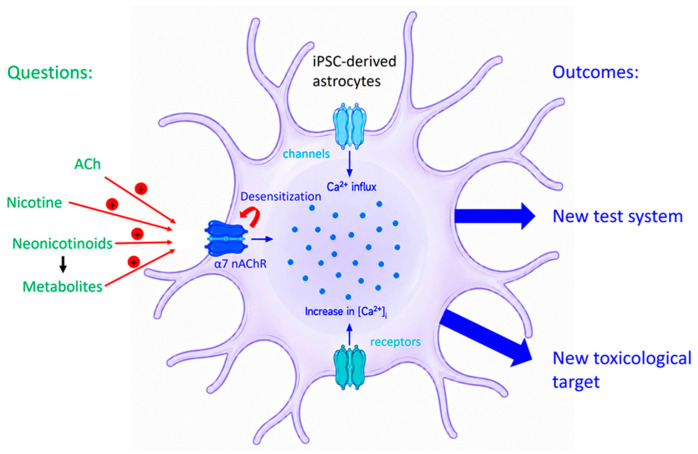
Graphical summary of main findings. Human iPSC-derived astrocytes respond to endogenous neurotransmitters, but also environmental toxicants such as neonicotinoid pesticides and their bioactive metabolites. Nicotine and neonicotinoids act through the α7 nAChR with a downstream involvement of voltage-gated cation channels leading to a change in [Ca^2+^]_i_. This provides an indication that astrocytes are a suitable test system for detecting acute functional effects of neurotoxicants, and have to be regarded as target cells for nicotinic compounds in the brain.

In this study (Figure 10), evidence is provided on nicotinic signalling in human iPSC-derived astrocytes. Data are provided on endogenous neurotransmitters, but also on environmental toxicants. A practical implication of this is that astrocytes may be used as a new test system to detect acute functional effects of neurotoxicants, like neonicotinoid pesticides and their bioactive metabolites. On the mechanistic level, we provide data that suggest that nicotine and neonicotinoid pesticides act on astrocytes mainly through the alpha7 nicotinic receptor, with a downstream involvement of voltage-gated cation channels. Evidence is provided here that the functional astrocytic response (increased intracellular calcium ion levels) is similar to that of human neurons. An implication of this finding for human toxicology is that astrocytes have to be regarded as target cells of nicotinic compounds in the brain.

## Data Availability

All data presented in manuscript figures are available in Excel files, so that other displays or statistical approaches may be applied to them. These data have been uploaded on Zenodo and can be accessed through the following link https://zenodo.org/records/20840279, accessed on 25 June 2026.

## Supplementary Materials

The following supporting information can be downloaded at: https://www.mdpi.com/article/10.3390/ijms27135902/s1. The supplementary data are found on https://zenodo.org/records/20840279, accessed on 25 June 2026.

## Abbreviations

The following abbreviations are used in this manuscript:

ACh	acetylcholine
ATP	adenosinetriphosphate
BMC_10_	Benchmark concentration 10
CaFFEE	Calcium fluorescent flash evaluating engine
C.I.	95% confidence interval
CYC	Cycloxaprid
DCT	descyanothiacloprid
DM	Differentiation medium
DN-IMI	Desnitro-imidacloprid
DNT	Developmental neurotoxicity
DoD	Day of differentiation
FPF	Flupyradifurone
GDNF	Glial-derived neurotrophic factor
GFAP	Glial fibrillary acidic protein
HTC	Highest-tested concentration
IMI	imidacloprid
iPSCs	Induced pluripotent stem cells
mAChR	Muscarinic acetylcholine receptor
nAChR	Nicotinic acetylcholine receptor
NeoNics	Neonicotinoids
NIC	Nicotine
Nif	Nifedipine
NMDA	N-methyl-D-aspartate
PBS	Phosphate buffered saline
PD	Parkinson’s disease
PI	Propidium iodide
PLO	Poly-L-ornithine
PM	Proliferation medium
THI	thiacloprid
Tubo	Tubocurarine
TTX	Tetrodotoxin
VDCCs	Voltage-dependent calcium channels
VGSCs	Voltage-gated sodium channels
VTD	Veratridine
