# Supplementary material for: Functional Expression of Nicotinic Receptors on iPSC-Derived Astrocytes and Signalling Disturbances by a Panel of Neonicotinoid Pesticides and Their Metabolites
Source: Int J Mol Sci. 2026 Jun 30;27(13):5902. doi: 10.3390/ijms27135902 (PMC13361451; doi:10.3390/ijms27135902)
Supplement: Supplementary file 1 [file ijms-27-05902-s001.zip › Supplementary material MDPI 1.pdf]

Supplementary methods

Antibodies

Table S1 Overview of used antibodies

Target	Species	Dilution	Supplier	Catalogue number	RRID
Primary					
NFkB p65	Rabbit	1:200	BioLegend	901301	AB_1151038
GFAP	Chicken	1:1000	Merck	AB5541	AB_177521
S100ß	Mouse	1:200	Sigma	S2532	AB_477499
Vimentin	Rabbit	1:200	abcam	Ab92547	AB_10562134
Secondary					
Goat anti-Mouse IgG1, Alexa Fluor 555		1:500	Invitrogen	A-21127	AB_2535769
Goat anti chicken 488		1:1000	Invitrogen	A-11039	AB_2534096
Donkey anit rabbit 647		1:1000	Invitrogen	A-31573	AB_2536183

Neurite outgrowth assay

The neurite outgrowth assay was performed as previously described [208-210]. In brief, cell culture dishes and flask were pre-coated with 50 µg/ml poly-l-ornithine (PLO) and 1 µg/ml fibronectin. Proliferating LUHMES cells were cultured in Advanced DMEM/F12 containing L-glutamine [2 mM], N2 supplement (1x), and FGF [40 ng/ml]. After reaching 80% confluency LUHMES cells were splitted and seeded at a density of 120,000 cells/cm^2 in a T75 flask in differentiation medium containing L-glutamine [2 mM], dibutyryl-cAMP [1 mM], tetracylcine [1 µg/ml], and GDNF [2 ng/ml]. After two days of differentiation (day 2), the cells were replated (103,000 cells/cm^2) into 96-well plates in differentiation medium containing L-glutamine [2 mM], tetracylcine [1 µg/ml], and GDNF [2 ng/ml]. One hour after replating, the cells were treated with a 10× concentrated stock solution of the test compound. Neurite outgrowth and viability were determined 24 h later, using automated high content imaging.

Neurite degeneration assay

The neurite degeneration assay was performed as previously described [209]. In brief, cell culture dishes and flask were pre-coated with 50 µg/ml poly-l-ornithine (PLO) and 1 µg/ml fibronectin. Proliferating LUHMES cells were cultured in Advanced DMEM/F12 containing L-glutamine [2 mM], N2 supplement (1x), and FGF [40 ng/ml]. After reaching 80% confluency LUHMES cells were splitted and seeded at a density of 120,000 cells/cm^2 in a T75 flask in differentiation medium containing L-glutamine [2 mM], dibutyryl-cAMP [1 mM], tetracylcine [1 µg/ml], and GDNF [2 ng/ml]. After two days of differentiation (day 2), the cells were replated (155,000 cells/cm^2) into 96-well plates in differentiation medium. Three days after replating on day 5, the cells were treated with a 10× concentrated stock solution of the test compound. Neurite area and viability were determined 24 h later, using automated high content imaging.

Image acquisition and quantification in neurite assays

For the neurite assays the image acquisition and analyses was performed as described previously [208,209]. In brief, cells were stained with Hoechst H-33342 [1 μg/ml] to visualise nuclei and calcein-AM [1 μM] to label viable cell structures. Fluorescent image acquisition was performed using an automated imaging system. For image analysis an algorithm detected the nuclei for defining the somatic regions. In a next step these areas were excluded from the total calcein-positice area. The remaining calcein-positive area was quantified as neurite area. Viability was determined by Hoechst and calcein-AM double positive cells compared to all detected nuclei.

Viability assessment of astrocytes

For viability assessment, astrocytes were seeded in 96-well plates as described above. Astrocyte viability was evaluated after 24 h incubation with the respective compound or solvent control. Astrocytes were stained with 1.8 μM Hoechst-33342 (H-33342; Merck, Darmstadt, Germany) and 1.5 μM propidium iodide (PI, Sigma-Aldrich, USA) for 1 h at 37 °C, 5% CO_2. Images were acquired using an ImageXpress Nano Automated Cell Imaging System (Molecular Devices, Sunnyvale, USA) with a 10x objective, capturing three random sites of each well (roughly 1000-1400 cells per site, 3000-4200 cells per well). H-33342 was imaged using the DAPI channel (Ex 377/50 nm, Em 447/60 nm), while PI was acquired via the TRITC channel (Ex 543/22 nm, Em 593/40 nm), with laser-based autofocus enabled. The "Cell scoring" module of MetaXpress (Molecular devices, Sunnyvale, USA) identified nuclei in the DAPI channel (min area: 6 μm^2, max: 22 μm^2). Cells were classified as viable when the PI staining intensity of the H-33342-positive area was negligible. Cell viability data are given as the percentage of viable cells, normalized to the solvent control. Triton X-100 (0.1% in PBS, 1 h; Sigma-Aldrich) served as positive control (>90% PI-positive cells). For analysis, at least two independent astrocyte differentiations were used, and images were taken from three technical replicate wells each. Solvent controls usually contained <10% cells staining positive for PI.

Intracellular ATP Measurement

For viability assessment, astrocytes were seeded in 96-well plates as described above. Astrocyte viability was evaluated after 24 h incubation with the respective compound or solvent control. After 24 h, a CellTiterGlo-TritonX-100 mix was prepared: a 0.5% Triton solution in PBS was prepared, and the commercial reagent mix CellTiterGlo 2.0 (Promgea, Madison, WI, USA) was added in a 1:1 ratio. A volume of 50 μL/well of the mix was added to each well (containing cells in 100 μL medium). The plate was shaken for two minutes, and 100 μL of the resultant cell lysate was transferred to a white measurement plate. For the blank value, only medium with the mix was measured. Luminescence data of samples were normalized to DMSO solvent controls, and the ATP data are given in % relative to "untreated cells".

Supplementary figures

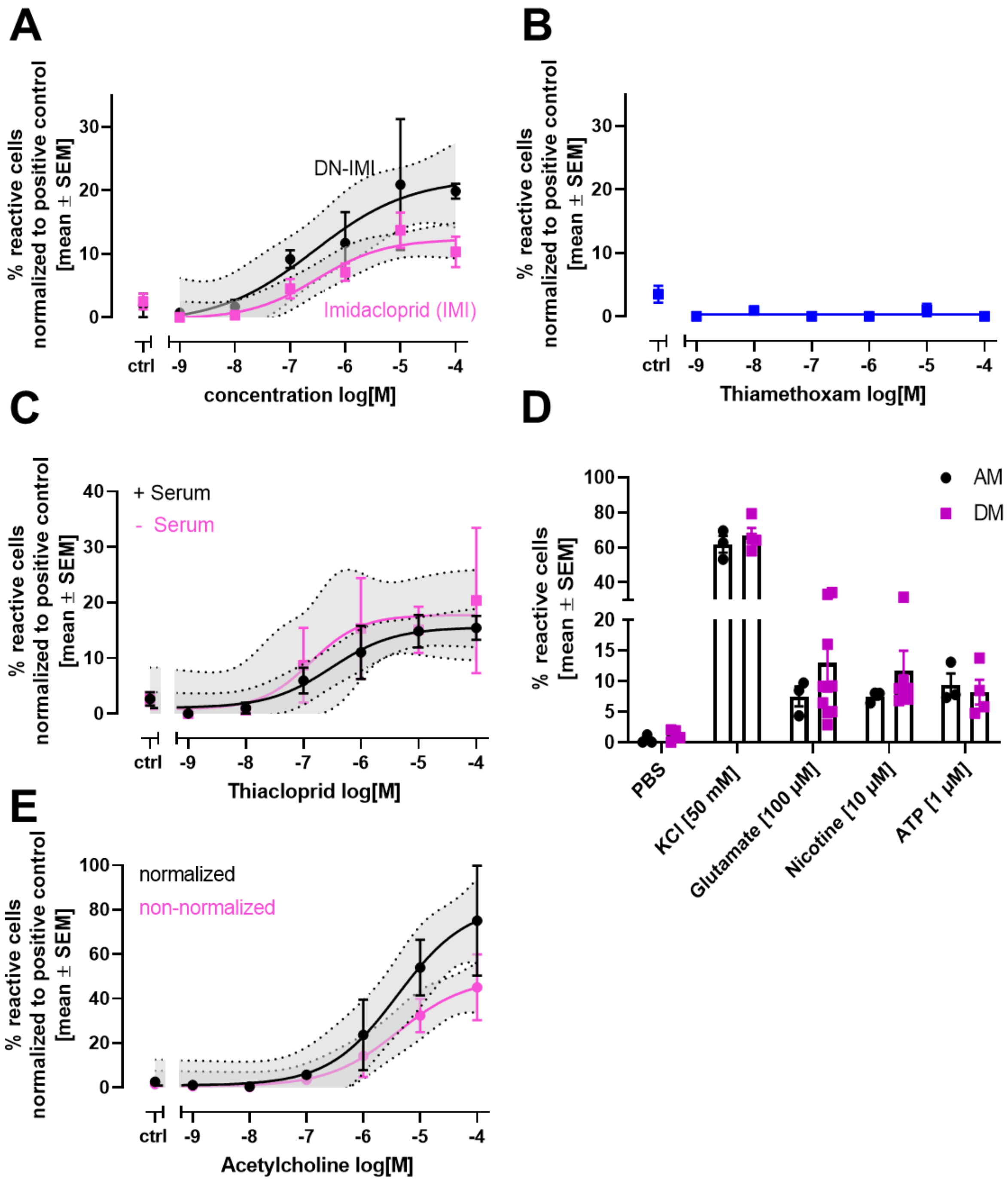

Figure S1: Response-specificity of astrocytes to some neonicotinoids

Astrocytes were used as in Fig. 2. Cells were stained 1 h before the experiment with a Ca^{2+} indicator dye. Compounds were added by an automated pipettor. Single-cell fluorescence information was recorded for the whole field of view. (A) Concentration-response curve of the parent imidacloprid (IMI) and its metabolite desnitro-imidacloprid (DN-IMI). This displays the behaviour of a well-known parent-metabolite pair, and thus complements data displayed in Fig. 1H for THI/DCT. (B) Concentration-response curve of Thiamethoxam. (C) Concentration-response curve of THI with and without 1%

Serum. (D) Receptor characterization of astrocytes cultivated in either astrocyte medium (AM) or differentiation medium (DM) after reseeding two days prior to calcium imaging.(E) Concentration-response curve of acetylcholine without normalization to number of responders in positive control and with normalization. Data is presented as % reactive cells normalized to the positive control for astrocytes. All data are mean ± SEM of biological triplicates, each biological replicate included 3 technical replicates. The 90% confidence intervals are indicated by dotted lines and grey background shading. Differences were tested for significance by two-way ANOVA, followed by Sidak ´s post hoc test for comparison between the conditions with and without serum. The gaussian distribution of the residuals was tested with four tests (Anderson-Darling, D'Agostino-Pearson omnibus, Shapiro-Wilk, Kolmogorvo-Smirnov). The results can the found in table 3 in the repository doi: 10.5281/zenodo.20840279.

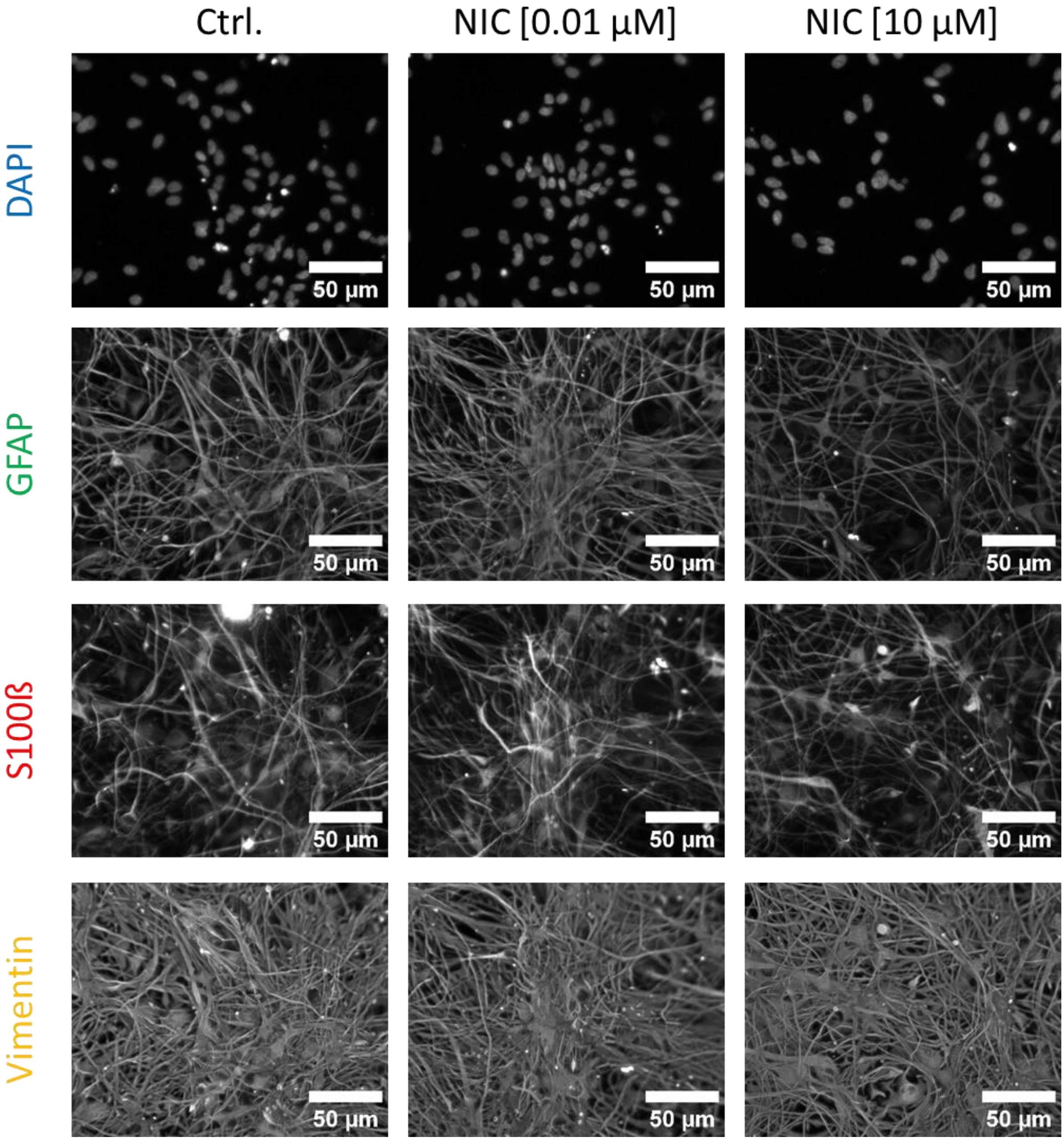

Figure S2: No obvious effects of long-term nicotine treatment on astrocyte phenotype

Expression of astrocyte specific marker (GFAP (green), S100ß (red), or Vimentin (yellow)) expression was investigated by immunohistochemistry after chronic NIC exposure for 7 days.

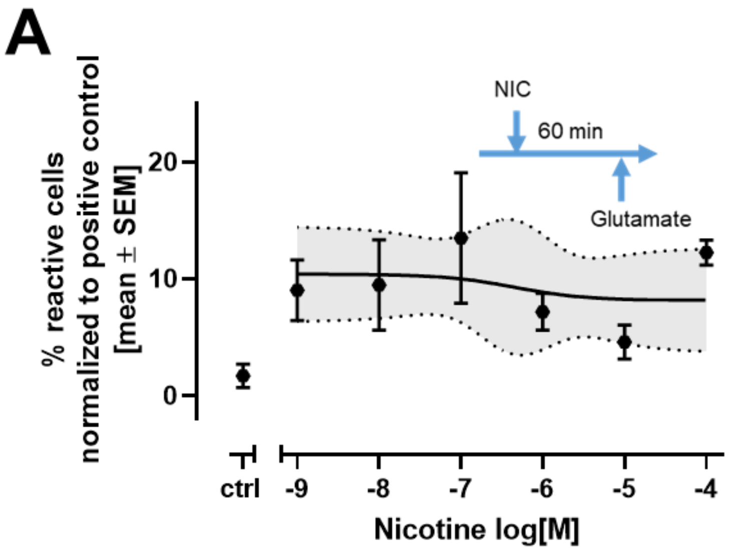

Figure S3: No obvious cross-desensitization of glutamate receptors upon nAChR activation

Concentration dependent response curves of a pre-treatment with NIC followed by stimulation with glutamate in hiPSC-derived astrocytes. Cells were stained 1 h before the experiment with a Ca^{2+} indicator dye. Compounds were added by an automated pipettor. Single-cell fluorescence information was recorded for the whole field of view. (A) Concentration response curve of Glutamate [100 µM] with a pre-treatment of NIC with concentrations from 0.001 to 100 µM. Data is presented as % reactive cells normalized to the positive control for astrocytes. All data are means ± SEM of biological triplicates, each biological replicate included 3 technical replicates. The 90% confidence intervals are indicated by dotted lines and grey background shading. Differences were tested for significance by two-way ANOVA, followed by Dunnett ´s multiple comparison post hoc test for comparison between treatments with the evoked rate of responders without pre-treatment of the stimulus. The gaussian distribution of the residuals was tested with four tests (Anderson-Darling, D'Agostino-Pearson omnibus, Shapiro-Wilk, Kolmogorvo-Smirnov). The results can the found in table 3 in the repository doi: 10.5281/zenodo.20840279.

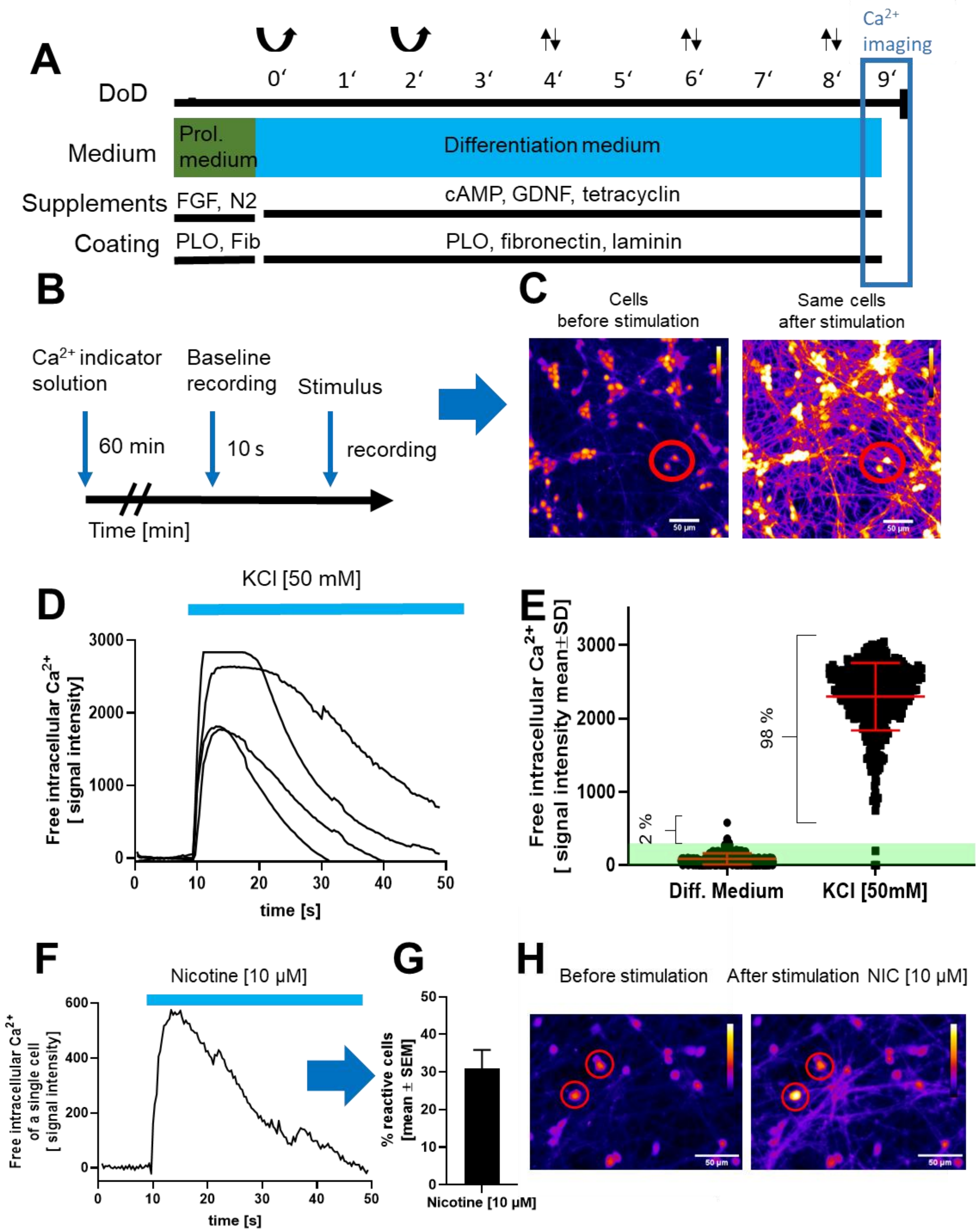

Figure S4: Use of LUHMES cells for single-cell calcium imaging

(A) Differentiation scheme for LUHMES cells: Cells were differentiated for 2 days (d2) in aT75 flask and then reseeded after two days into 96-well plates at a density of 220,000 cells/cm² in LUHMES differentiation medium (DM) (with cAMP, tetracycline, and glial cell line-derived neurotrophic factor (GDNF)). Half a medium exchange was performed every other day until day 9. On day 9 calcium imaging experiments were performed. (B) Experimental set-up for the calcium

imaging experiments. LUHMES cells were plated in 96-well plates and cultured for 7 days. At 1 h before the experiment cells were loaded with a Ca^{2+} indicator dye. Test compounds were added by an automated pipettor. Single-cell fluorescence information was recorded for the whole field of view. (C) Exemplification of calcium fluorescence images of day 9 LUHMES cells before and after stimulation with KCl [50 mM]. (D) Individual cell somata were automatically identified by the CaFFEE software and single-cell calcium traces were obtained after stimulation with KCl [50 mM]. (E) Threshold determination for the cell population to define a 'reactive cell': The average fluorescence (means ± SD) was determined for about 1000 cells. The threshold of 'reactivity' was set at, 'means + 3x the standard deviation' of negative controls. Only 2% of solvent-treated cells exceeded that value. Typical data for about 500 positive controls are shown. (F) Exemplification of a calcium fluorescence trace of day 9 LUHMES cells after stimulation with nicotine (NIC) [10 μM]. (G) Quantification of the amount of reactive cells in % after the stimulation with NIC [10 μM]. (H) Exemplification of a calcium fluorescence images of day 9 LUHMES cells before and after stimulation with nicotine (NIC) [10 μM]. Data is presented as % reactive cells, normalized to the number of positive control cells as mean ± SEM of biological triplicates, each biological replicate included 3 technical replicates.

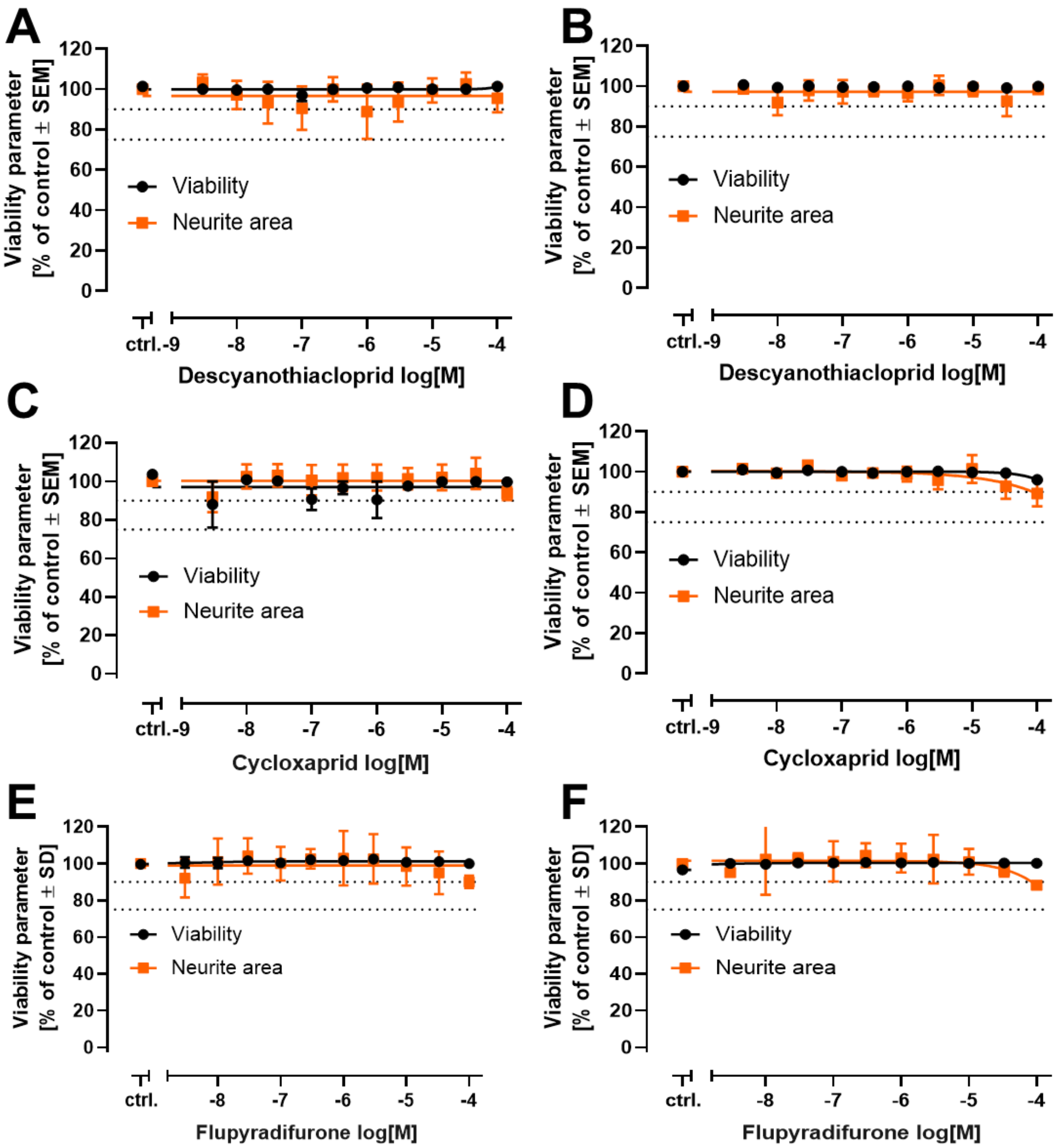

Figure S5: Absence of neurite toxicity triggered by DCT and the neonicotinoid pesticide CYC, FPF

(A, C, E) Neurite area and viability assessment in the neurite degeneration assay for the compounds DCT, CYC, FPF in LUHMES cells. (B, D, F) Neurite area and viability assessment in the neurite outgrowth assay for the compounds DCT, CYC, FPF in LUHMES cells. Providing evidence that the observed effect in Fig. 8B is not due to cytotoxicity. Data is presented as % relative to control, as mean ± SEM of biological triplicates, each biological replicate included 3 technical replicates.

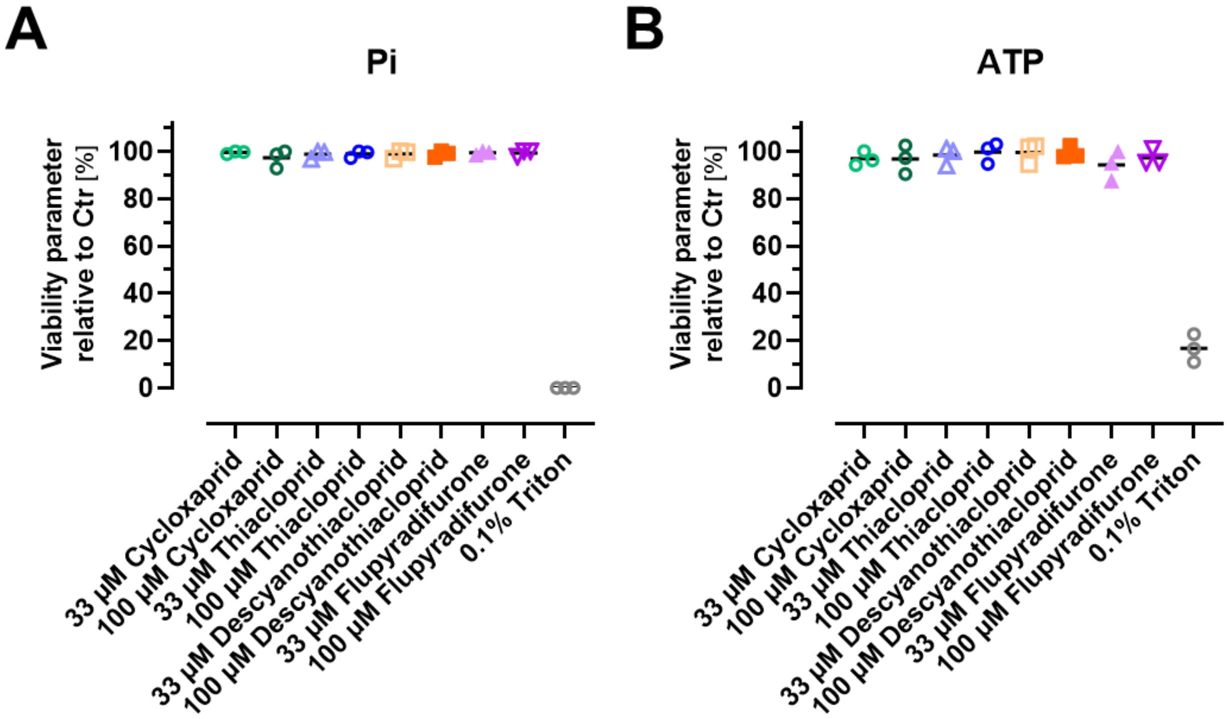

Figure S6: Viability assessment of iPSC-derived astrocytes after NeoNic treatment

Astrocytes (iPSC-derived) were cultured and used as in Fig. 2. Viability was assessed 24 h after compound treatment comparably to the dead control 0.1 % Triton. (A) Cell viability assessment by PI staining 1h after staining. (B) Cell viability assessment measuring ATP content of astrocytes. Data is presented as % relative to control, single data points of each biological triplicate are shown, each biological replicate included 3 technical replicates.

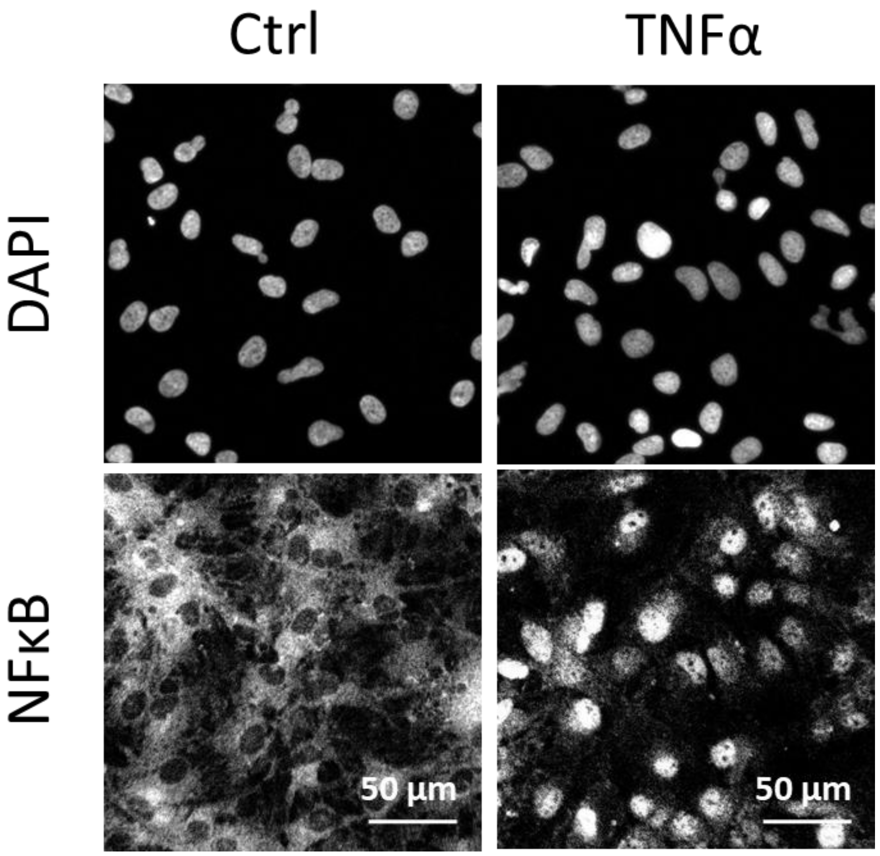

Figure S7: TNFα-induced NFκB translocation in iPSC-derived astrocytes

Astrocytes (iPSC-derived) were cultured and used as in Fig. 2, but experiments involved a pre-incubation step to clarify whether nicotine affected inflammatory TNFα responses or TNFα affected nicotinic Ca2+ responses. Exemplary images of nuclear stain with H-33342 and astrocytic NFκB distribution. Unstimulated astrocytes (Ctrl) had virtually all NFκB stain in their cytosol. The position of the nuclei is obvious from oval black holes. The TNFα stimulated astrocytes had virtually no cytosolic NFκB signal, while the nuclear area stained intensely.
