# Supplementary figures and images for: Functional Expression of Nicotinic Receptors on iPSC-Derived Astrocytes and Signalling Disturbances by a Panel of Neonicotinoid Pesticides and Their Metabolites
Source: Int J Mol Sci. 2026 Jun 30;27(13):5902. doi: 10.3390/ijms27135902 (PMC13361451; doi:10.3390/ijms27135902)

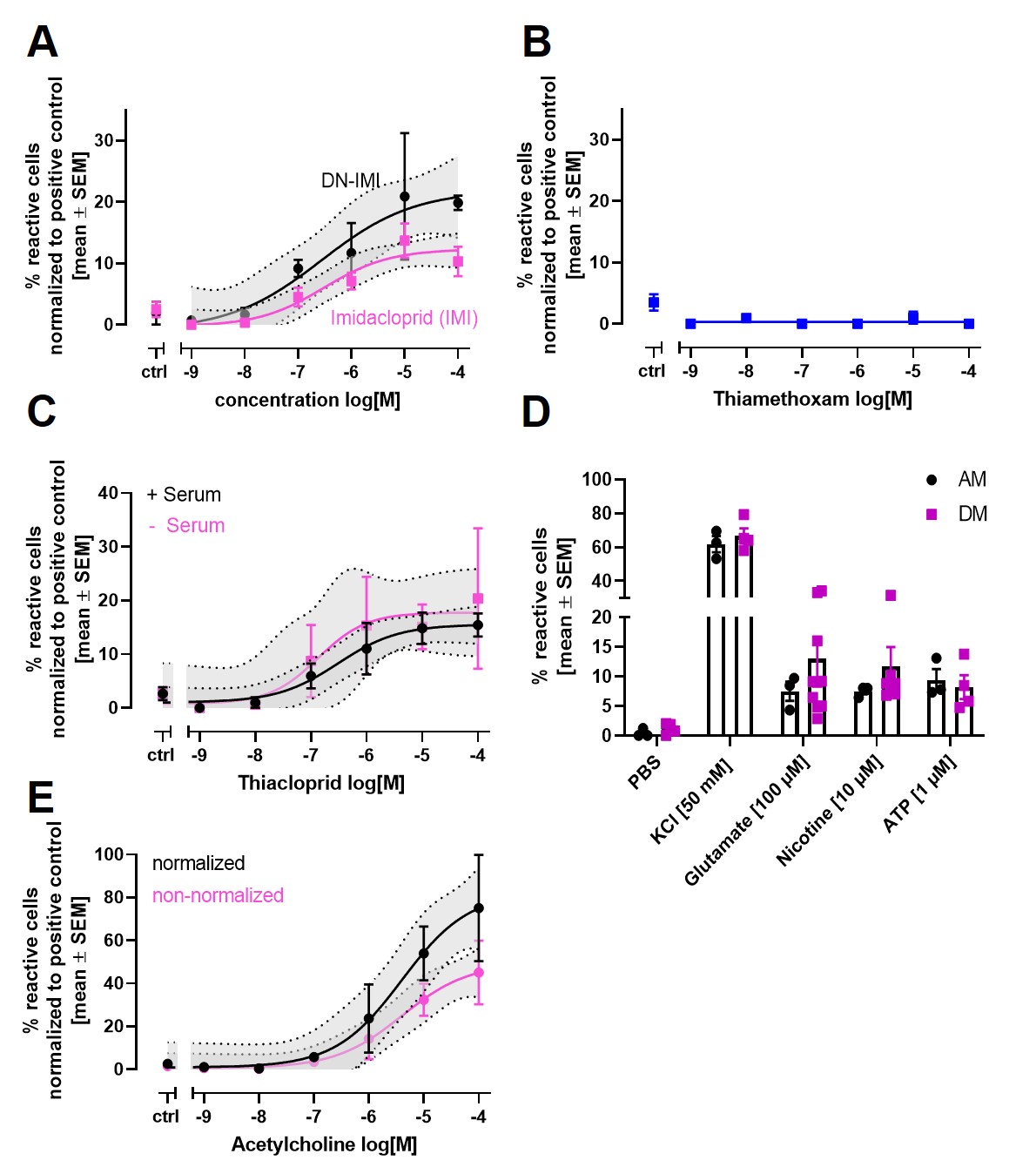

Supplement: Supplementary file 1 [file ijms-27-05902-s001.zip › Supp. Figure S1.jpg]

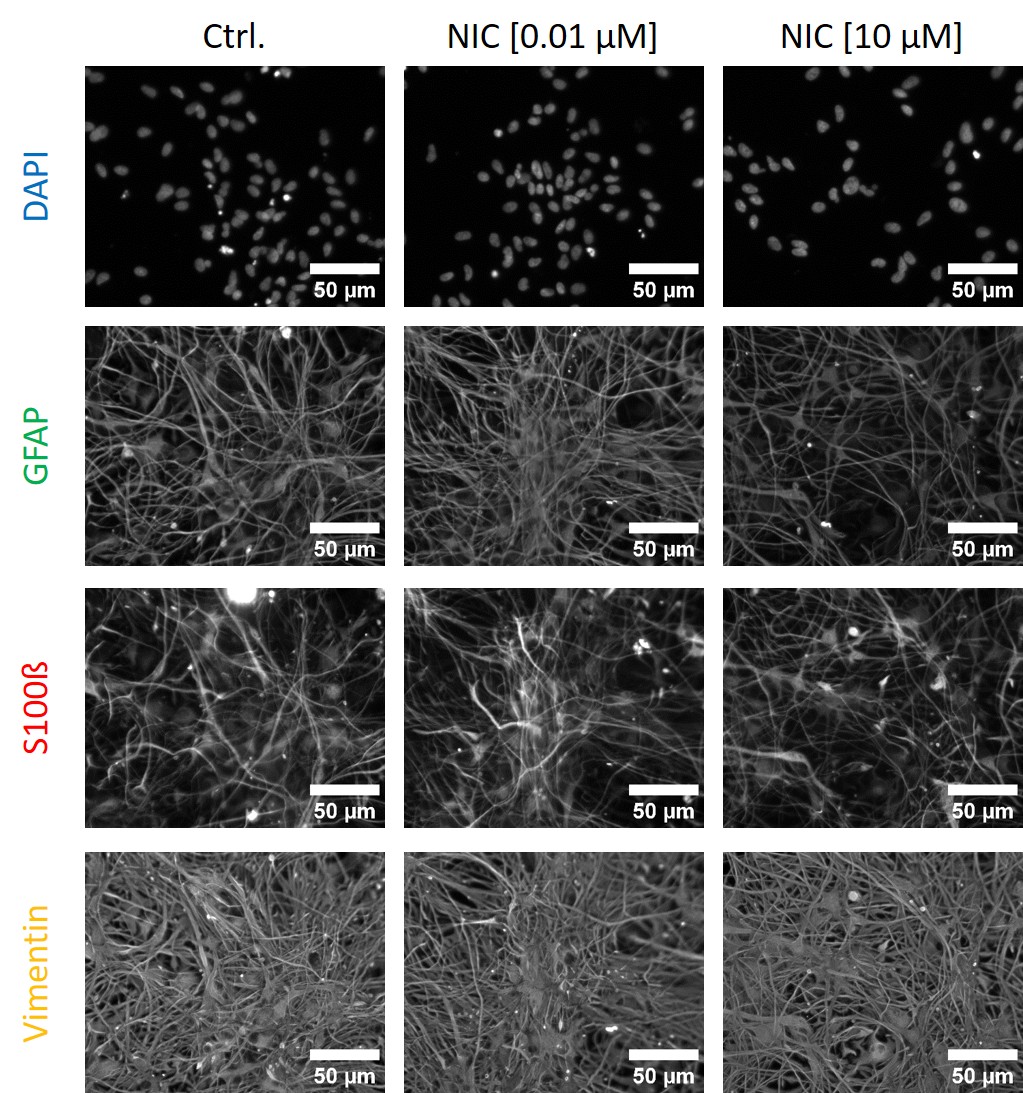

Supplement: Supplementary file 1 [file ijms-27-05902-s001.zip › Supp. Figure S2.jpg]

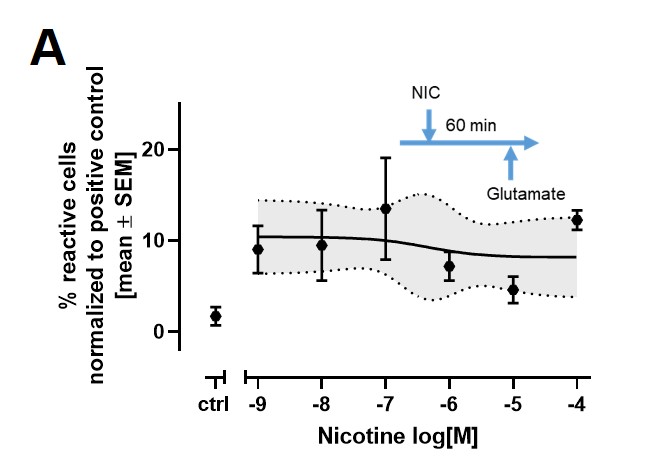

Supplement: Supplementary file 1 [file ijms-27-05902-s001.zip › Supp. Figure S3.jpg]

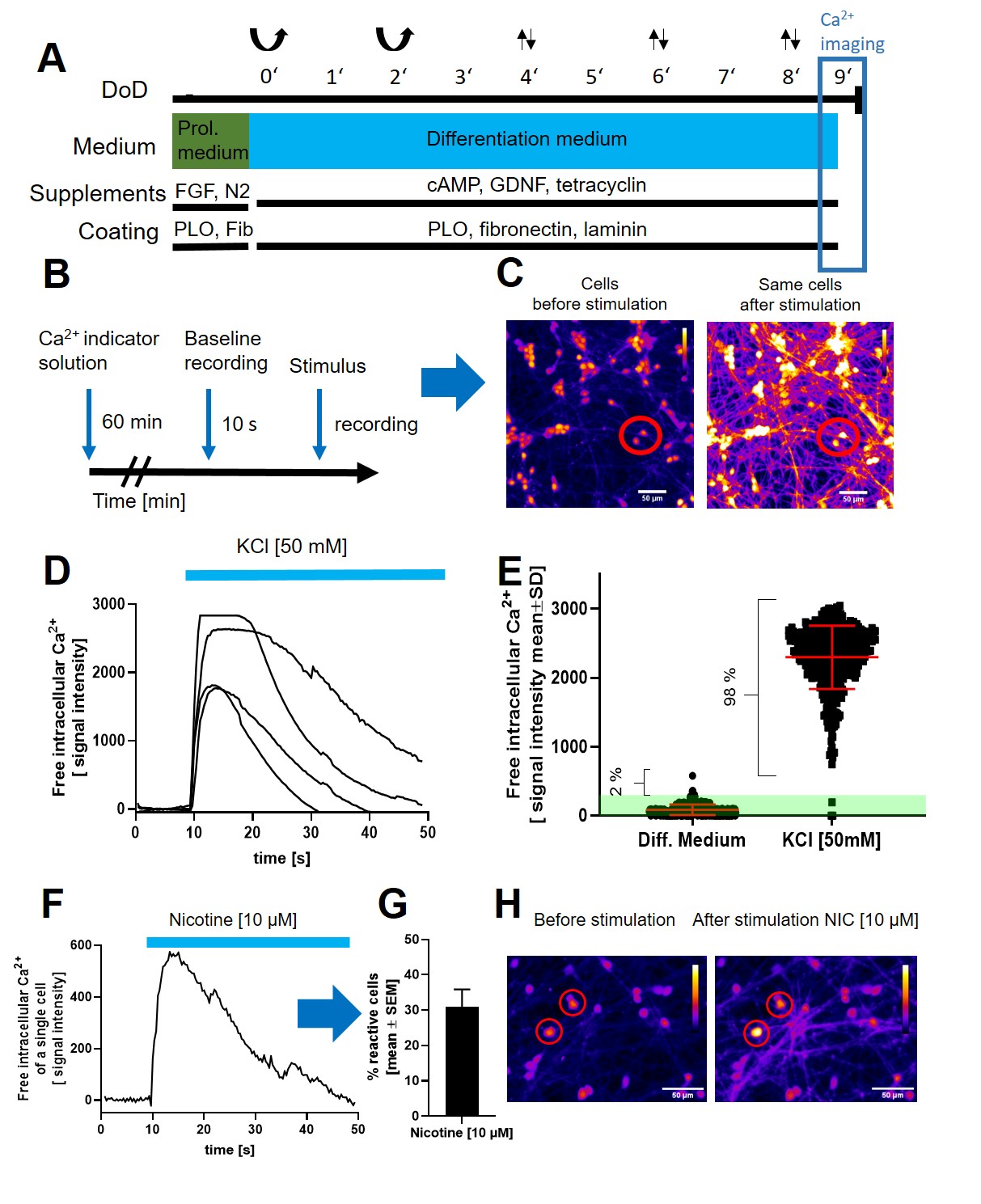

Supplement: Supplementary file 1 [file ijms-27-05902-s001.zip › Supp. Figure S4.jpg]

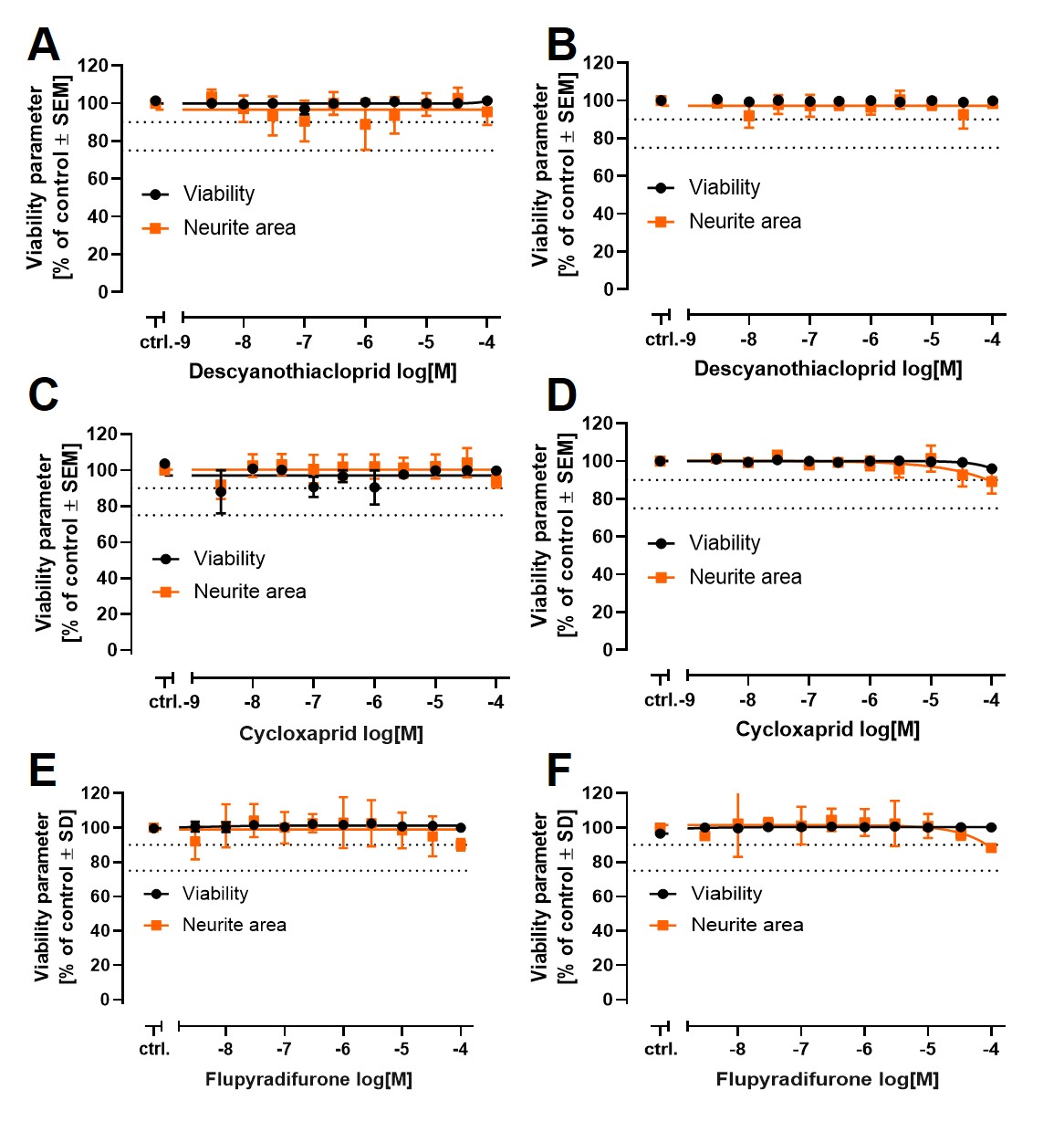

Supplement: Supplementary file 1 [file ijms-27-05902-s001.zip › Supp. Figure S5.jpg]

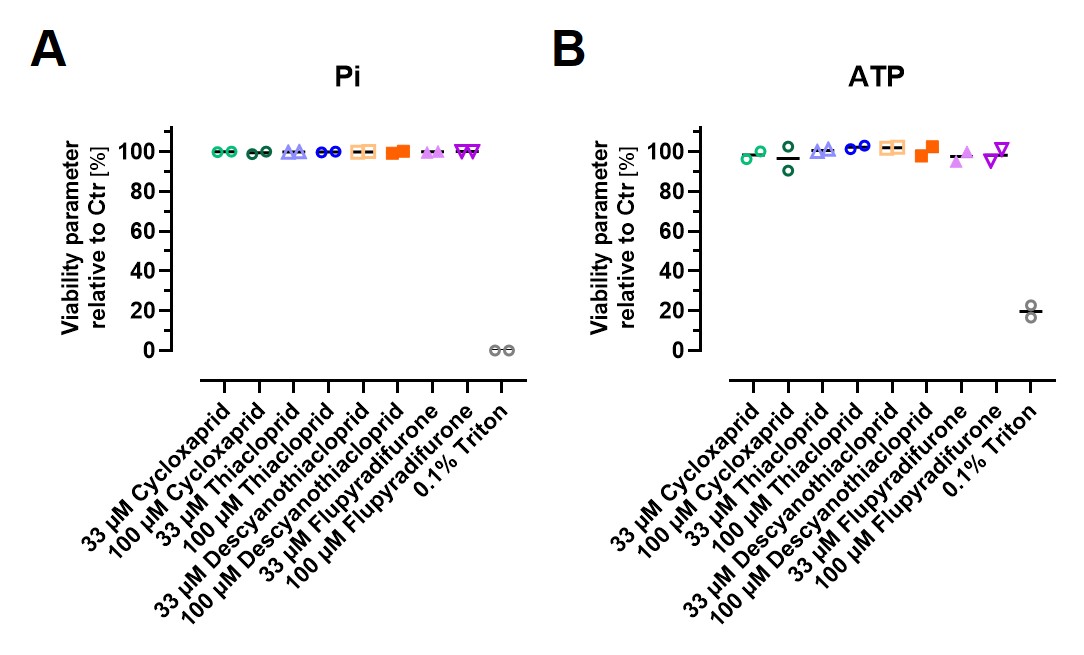

Supplement: Supplementary file 1 [file ijms-27-05902-s001.zip › Supp. Figure S6.jpg]
